# New insights into the structure-based mechanism of *Bacillus subtilis* spore resistance to high hydrostatic pressure

**DOI:** 10.1128/aem.00070-26

**Published:** 2026-04-27

**Authors:** Shengnan Kang, Ziqi Gong, Junyi Zhang, Qiuyu Meng, Jinyang Li, Fengzhi Lyu, Lei Rao, Xiaojun Liao

**Affiliations:** 1 College of Food Science and Nutritional Engineering, National Engineering Research Center for Fruit and Vegetable Processing, Key Laboratory of Fruit and Vegetable Processing of the Ministry of Agriculture and Rural Affairs, Beijing Key Laboratory for Food Non-Thermal Processing, China Agricultural University 630105 , Beijing, China; University of Georgia Center for Food Safety, Griffin, Georgia, USA

**Keywords:** *Bacillus subtilis *spore, structural modification, high hydrostatic pressure, germination, pressure resistance

## Abstract

The exceptional pressure resistance of bacterial spores limits the application of high hydrostatic pressure (HHP) in food sterilization, yet the underlying mechanisms remain poorly understood. Here, we examined how structural modifications (coat, cortex, inner membrane [IM], α/β-type small acid-soluble proteins [α/β-type SASPs]) in *Bacillus subtilis* spores affect HHP-induced germination, superdormant (SD) subpopulation, and inactivation, which together define HHP resistance. Key findings reveal that (i) coat defects likely alleviate cortex compression on IM, thereby influencing germination-associated proteins. This alteration reduces germination efficiency at 200 MPa but enhances it at 500 MPa, leading to an expanded SD subpopulation and increased resistance at 200 MPa, while a diminished SD ratio and reduced resistance at 500 MPa; (ii) increased cortex cross-linking combined with core expansion potentially modifies IM properties, strongly affecting germination-related proteins. This change markedly promotes germination under both 200 and 500 MPa, resulting in decreased SD ratios and thus lower spore resistance under both conditions; (iii) reduced cardiolipin content in IM likely alters its biophysical properties, specifically increasing germination efficiency at 500 MPa. This contributes to a lower SD ratio and reduced resistance at 500 MPa; (iv) the absence of α/β-type SASPs has minimal effect on resistance at 500 MPa but reduces resistance at 200 MPa by decreasing the SD ratio, potentially due to spontaneous germination after treatment. Collectively, HHP resistance is modulated by multiple structural components through distinct mechanisms. Structural modifications that alter IM biophysical properties or the function of germination-related proteins appear to be critical regulators of pressure-induced germination.

**IMPORTANCE:**

Our findings elucidate the structural mechanism underlying the exceptional pressure resistance of bacterial spores, which represent a major challenge for high hydrostatic pressure sterilization. This study establishes a structure-based framework that not only clarifies the biophysical principles of spore resilience but also provides a foundation for developing novel strategies to achieve mild spore inactivation in the food industry. Beyond these practical implications, our work offers fundamental theoretical insights into microbial pressure resistance mechanisms.

## INTRODUCTION

**Fig 1:**
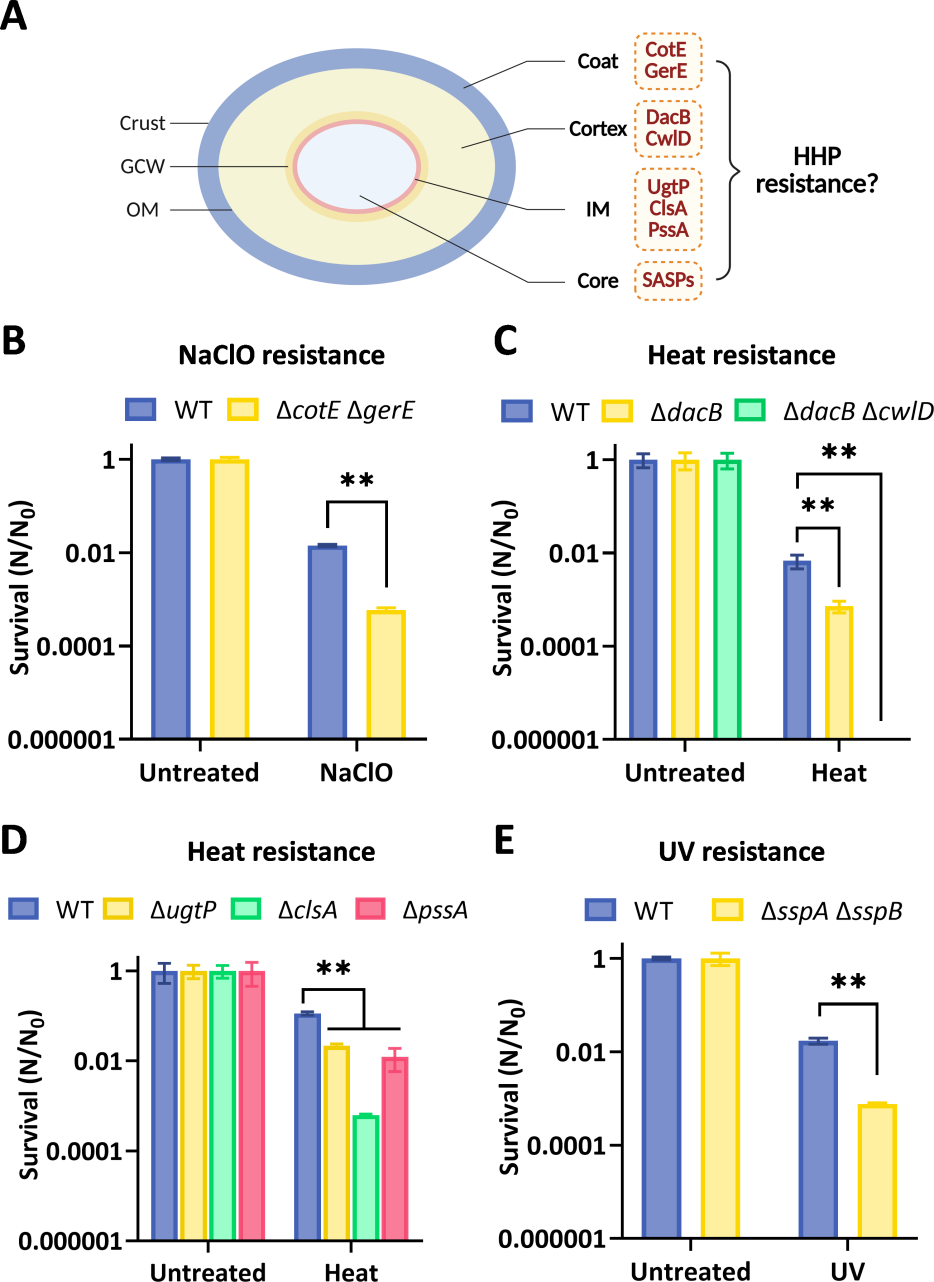
The effects of structural modifications of *Bacillus subtilis* spores on their resistance to various treatments. (**A**) Overview of spore structures and associated proteins: CotE is essential for the assembly of many coat proteins and the outer coat layer (22). GerE regulates gene transcription in the mother cell during sporulation, particularly controlling the final stages of coat deposition and maturation, thereby producing spores with enhanced resistance (23). DacB determines the degree of cortex peptidoglycan (PG) cross-linking (24, 25). CwlD is a muramidase involved in the formation of muramic-δ-lactam in the cortex PG and is critical for cortex-lytic enzyme (CLE) recognition and cortex degradation (26). UgtP is required for the synthesis of neutral diglucosyl-1,2-diacylglycerol (dGDG), an important membrane anchor for lipoteichoic acids (27). ClsA catalyzes the synthesis of cardiolipin (CL) (28). PssA is primarily responsible for synthesizing phosphatidylethanolamine (PE) (29). α/β-type small acid-soluble proteins (SASPs) protect spore DNA from damage caused by moisture, heat, chemicals, and radiation (30, 31). (**B**) NaClO resistance assay: wild-type (WT) and coat-defective spores (Δ*cotE* Δ*gerE*) were exposed to 2.5% (0.34 M) NaClO at 23°C for 5 min, and the spores were neutralized with 0.1 M sodium thiosulfate. (**C and D**) Heat resistance assay: (**C**) WT and cortex-modified spores (Δ*dacB*, Δ*dacB* Δ*cwlD*); (**D**) WT and IM-modified spores (Δ*ugtP*, Δ*clsA*, and Δ*pssA*) were treated at 93°C for 10 min. (**E**) UV resistance assay: WT and DNA-protecting proteins-modified spores (Δ*sspA* Δ*sspB*) were irradiated with a 254 nm, 30 W lamp at a distance of 45 cm. All experiments were performed in triplicate, and data are presented as the mean ± SD of three independent replicates. **P < 0.01.

Spores represent a dormant cellular state formed by spore-forming bacteria, including *Bacillus* and *Clostridium* species, in response to unfavorable environmental conditions, such as nutrient deprivation (1, 2). *Bacillus subtilis* has been established as a principal model organism for the study of bacterial sporulation and germination (3). Dormant spores of *B. subtilis* exhibit a highly specialized, multi-layered architecture, comprising—from exterior to interior—the crust, coat, outer membrane, cortex, germ cell wall, inner membrane (IM), and core (Fig. 1A) (4–7). Each structural layer contributes distinct protective functions: the coat serves as a molecular sieve or reactive barrier to prevent or detoxify harmful chemicals, the cortex is critical for heat resistance, and the core is associated with resilience to UV radiation and high temperatures (5, 6, 8–10). These structures collectively confer extraordinary resistance, enabling spores to remain viable for centuries or longer (5, 11–13). Given their ubiquity in natural environments, spores can readily enter the food supply chain, posing risks of spoilage and foodborne disease (14–16). Conventional sterilization techniques, such as pasteurization (<75°C), are often insufficient for spore inactivation (17, 18), whereas more aggressive thermal treatments (121°C–135°C), though effective, can degrade food quality (19–21). Consequently, it is of great significance to develop novel technologies capable of achieving effective spore inactivation while preserving the sensory and nutritional attributes of food.

High hydrostatic pressure (HHP) is a non-thermal food processing technology effective in inactivating vegetative cells of pathogenic and spoilage microorganisms, with the advantage of minimizing heat-induced quality degradation in food products (32, 33). However, the inherent extreme resistance of bacterial spores to HHP poses a significant limitation to its application in low-acid foods (34). While HHP alone is generally insufficient to directly inactivate spores, it can induce spore germination—a physiological transition that renders spores susceptible to milder subsequent treatments (35, 36). This principle has motivated the development of a “germination-inactivation” strategy for spore control in HHP processing (37). HHP-induced spore germination proceeds through two distinct pressure-dependent pathways: the moderate high pressure (MHP; 50–300 MPa) pathway and the very high pressure (VHP; 400–600 MPa) pathway (37, 38). Under MHP conditions, germination is initiated by the activation of GerA-type germinant receptors (GRs) located in the IM, leading to an irreversible commitment to germinate and triggering the release of Ca^2+^-dipicolinic acid (Ca-DPA) through the SpoVA channel (37, 39). In contrast, VHP bypasses GR-mediated signaling by directly opening SpoVA channels, resulting in rapid Ca-DPA release (40, 41). Following Ca-DPA excretion, spore germination advances through water uptake, activation of cortex-lytic enzymes (CLEs), degradation of the cortex, core rehydration, and ultimately the loss of spore-specific resistance properties (36, 38).

High germination efficiency in bacterial spores is a prerequisite for the successful application of the “germination-inactivation” strategy. Spore germination efficiency is strongly influenced by spore structure. Previous studies have indicated that environmental conditions during sporulation—such as temperature, pH, and medium composition—can significantly alter spore architecture, including the coat, cortex, IM, and core, thereby modulating the germination behavior of the future spores (42, 43). Moreover, genetic modifications that alter IM lipid composition have been shown to affect germination efficiency (29). These observations suggest that structural variations may also influence HHP-induced germination, although direct evidence remains limited. Similarly, spore resistance to HHP is closely linked to structural integrity. As noted earlier, spore layers confer protection against diverse stressors, including chemical agents, UV radiation, and heat (10). A recent study further demonstrated that coat defects reduce HHP resistance in germinated spores with an intact cortex, while cortex degradation markedly diminishes spore resistance and enhances inactivation under HHP (44). This implies that other structural components may also contribute to HHP resistance, though systematic investigation is still needed. Furthermore, our previous work identified the presence of high-pressure superdormant (HPSD) spores that survive HHP treatment (45, 46). These spores can undergo spontaneous germination during post-incubation, thereby improving the overall sporicidal efficacy of the HHP-based germination-inactivation strategy (46). Understanding the factors that regulate the spontaneous germination of HPSD spores and elucidating the underlying mechanisms could therefore advance the development of more effective spore inactivation methods using HHP. In fact, external factors, such as HHP treatment intensity and post-incubation conditions, have been shown to influence HPSD spore germination (46). However, the role of intrinsic structural features of spores in modulating this process remains largely unexplored.

In this study, we systematically investigate the contribution of key structural components of bacterial spores to their germination and inactivation under HHP. A series of mutant strains with targeted modifications in critical structures—including the coat, cortex, IM lipid composition, and DNA protective proteins—were constructed, and the resulting spores were subjected to HHP treatment. The germination kinetics, survival rates, and superdormant (SD) ratios of the mutant spores were quantitatively assessed. Our findings demonstrate that the integrity of the coat, modification in cortex or IM significantly affects HHP-induced germination and inactivation, while alterations in α/β-type small acid-soluble proteins (SASPs) had minor effects. These results provide new insights into the mechanisms of bacterial spore response to HHP and establish a theoretical foundation for optimizing HHP-based sterilization strategies in the food industry.

## MATERIALS AND METHODS

### *Bacillus subtilis* strains and spore preparation

All *B. subtilis* strains utilized in this study are listed in Table S1. Mutant alleles were constructed in the PS832 (wild type) or bLA201 (Δ*5*, deficient in all GerA-type germinant receptors: Δ*gerBB*, Δ*gerKB*, Δ*yfkT*, Δ*yndE*, Δ*gerA*) backgrounds using a gene replacement strategy (47). Two mutagenesis strategies were employed. The first involved amplifying the flanking regions of the target genes and assembling them with an antibiotic resistance cassette *in vitro* using Gibson Assembly Master Mix (New England Biolabs, USA). Alternatively, a direct cloning method was used, whereby the entire antibiotic resistance cassette, along with its extended upstream and downstream genomic regions, was amplified as a single fragment from an existing mutant strain. This PCR product was then directly used to transform the target *B. subtilis* strain, achieving allele replacement via homologous recombination.

The spores used in this study were prepared following a previously described method with some modifications (48). Wild-type (WT) and mutant strains were grown in Luria Bertani Broth (LB, Oxoid, England) at 37°C overnight. Sporulation was induced by transferring the overnight-cultured cells into 2 L of Schaeffer’s liquid medium (Difco Sporulation Medium, DSM) and incubating at 37°C for 32 h. The spores were harvested by centrifugation (9,168 × *g*, 4°C, 10 min) and washed three times with double-distilled water (DDW). To eliminate residual vegetative cells, the spore suspensions were subjected to daily washing with DDW for seven consecutive days, followed by purification via buoyant density centrifugation using a Nycodenz gradient. Specifically, spores were resuspended in 20% Nycodenz, layered over 900 µL of 50% Nycodenz solution, and centrifuged (20,627 × *g*, 4°C, 20 min). The supernatant was carefully discarded, and the purified spore pellet was washed extensively with DDW (at least five times). Phase-contrast microscopy confirmed that the final preparation contained >99% phase-bright spores. The purified spores were resuspended in DDW and stored at 4°C until use.

### Measurement of spore resistance

Spores were adjusted to OD_600_ = 0.5 in DDW for resistance measurement. The resistance of spores to various stressors was evaluated using the following treatments: (i) 2.5% (0.34 M) NaClO treatment (Huada, China) at 23°C for 5 min. After treatment, the spores were neutralized with 0.1 M sodium thiosulfate (Hongmeng, China); (ii) metal bath wet heat treatment at 93°C for 10 min and then immediately placed on ice until testing (MIULAB, China); and (iii) UV irradiation treatment using a UV lamp at 254 nm, 30 W, and a 45 cm distance (Jinlin, China). The protocols for chemical and UV treatments were adapted from previously described methods (45, 49, 50). Following each treatment, samples were serially diluted 10-fold in sterile phosphate-buffered saline. A 100 µL aliquot of each dilution was spread onto LB agar plates, followed by overnight incubation at 37°C. Colonies were enumerated once no further emergence of new colonies was observed, with a detection limit of 10 colony-forming units (CFU)/mL. All experiments were performed in triplicate assays of three independent spore preparations for each strain.

### Measurement of spore germination

Spore germination was assessed under nutrient and non-nutrient conditions. For nutrient-induced germination, spores were first heat-activated at 75°C for 30 min and then cooled on ice for 15 min. Unless otherwise stated, spores were adjusted to OD_600_ = 0.5 in 25 mM K-Hepes buffer with 50 μM TbCl_3_ and various germinants. Germination was initiated with one of the following germinants: (i) 10 mM L-alanine; (ii) AGFK (5 mg/mL D-glucose, 5 mg/mL D-fructose, 50 mM KCl, 2.5 mM L-asparagine); (iii) 1 mM dodecylamine (DDA). For HHP-triggered germination, spores are suspended in DDW (OD_600_ = 0.5) and treated by (iv) 200 MPa at 30°C for 1–10 min; and (v) 500 MPa at 30°C for 1–10 min. After treatment, spore suspensions are centrifuged (9,168 × *g*, 4°C, 5 min), and 198 μL supernatant is mixed with 2 μL TbCl_3_ (5 mM) to test DPA release. For spontaneous germination, spores (OD_600_ = 2) were treated with 500 MPa for 3 min at 25°C and centrifuged (9,168 × *g*, 4°C, 5 min) to detect DPA in the supernatant. Spores were resuspended to an OD_600_ = 0.5 and incubated at 37°C for monitoring. Notably, non-nutrient germinants (DDA and HHP) were applied without prior heat activation, as previously described (51).

Germination kinetics were monitored by CaDPA release, an early indicator of spore germination (52), using Tb^3+^-DPA fluorescence (excitation 270 nm, emission 545 nm) measured on a Spark 10 M plate reader (Tecan, Switzerland). For phase-contrast microscopy, 20 µL aliquots of spore suspensions were centrifuged, and the pellets were resuspended in 5–10 µL of DDW. Images were captured using a Nikon DS-Qi2 microscope equipped with a Nikon Plan Apo Lambda 100×/1.45 oil immersion objective. For each condition, at least three independent fields of view were examined, encompassing a minimum of 300 spores per replicate to ensure statistical reliability. Representative images are presented to illustrate the findings. All experiments were performed in triplicate assays of three independent spore preparations for each strain.

### The decoating procedure for spores

Dormant spores (OD_600_ = 50) were subjected to decoating in a decoating buffer (50 mM Tris-HCl [pH 7.4], 8 M urea, 1% SDS, 50 mM DTT) at 37°C for 2 h with constant rotation (53). Following incubation, the suspension was centrifuged (20,627 × *g*, 4°C, 5 min). The pellet was collected and washed thoroughly with DDW for at least five cycles. The decanted spores were resuspended in 0.85% NaCl solution containing 25 mg/mL lysozyme and incubated at 37°C for 20 min. Successful decoating was confirmed by monitoring the decrease in OD_600_ during germination and by phase-contrast microscopy. Finally, the decanted spores were resuspended in DDW and stored at 4°C until use.

### Measurement of viability and superdormancy of spores after HHP treatment

To assess spore viability after HHP treatment, spores were subjected to 200 or 500 MPa at 30°C for 10 min, and survival was quantified by CFU counts. For the assay of SD spores, a protocol adapted from Zhang et al. was employed (46). Following HHP treatment, spores were incubated at 37°C/2 h for spontaneous germination and then pasteurized at 80°C for 20 min. For both viability and SD spore assays, the treated samples were serially diluted 10-fold in DDW, and 100 μL aliquots of the appropriate dilutions were spread-plated onto LB agar plates. The plates were incubated overnight at 37°C, and colonies were enumerated until no further growth was observed. The limit of detection for this assay was 10 CFU/mL. All experiments were performed in triplicate assays of three independent spore preparations for each strain.

### Data processing

All experiments were conducted with three independent replicates. Data were analyzed using one-way analysis of variance, followed by Duncan’s multiple range test in SPSS (Version 26.0; IBM Corp., Armonk, NY, USA). Differences between treatments were considered statistically significant at *P < 0.05, **P < 0.01, and ***P < 0.001. All figures were generated using GraphPad Prism (Version 9.5.0 for Windows; GraphPad Software, San Diego, CA, USA).

## RESULTS

### Construction of mutant strains targeting genes involved in spore coat, cortex, inner membrane, and DNA protection

To investigate the effects of spore intrinsic structure on HHP-induced germination, we constructed multiple mutant strains by targeting genes associated with the spore coat, cortex, inner membrane (IM), and DNA protection protein (Fig. 1A; Table S1). Successful construction of each mutant was verified through established resistance assays (Fig. 1B through E). Specifically, the NaClO resistance assay was applied to confirm the successful construction of coat mutant strains (22, 54). As expected, the Δ*cotE* Δ*gerE* spores exhibited a significantly lower survival rate than WT spores (Fig. 1B), confirming the impairment of coat integrity. Similarly, heat resistance assay was employed to validate mutants with alterations in cortex or IM (29, 55, 56). As shown in Fig. 1C and D, cortex- (Δ*dacB* and Δ*dacB* Δ*cwlD*) and IM-modification (Δ*ugtP*, Δ*clsA*, and Δ*pssA*) spores showed markedly reduced survival under heat stress compared to WT, confirming successful mutagenesis. Moreover, UV resistance was utilized to verify mutant spores with lowered DNA protection capacity (57, 58). As shown in Fig. 1E, mutant spores lacking DNA protection proteins (Δ*sspA* Δ*sspB*) exhibited significantly reduced UV resistance, confirming successful strain generation. Additionally, all aforementioned mutations were also introduced into a Δ*5* strain background, which lacks all GerA-type GRs (Δ*gerBB*, Δ*gerKB*, Δ*yfkT*, Δ*yndE*, and Δ*gerA*) (59). The phenotypes of the corresponding mutant spores were also validated accordingly (Fig. S1).

### The germination behavior of coat-defective spores under HHP

**Fig 2:**
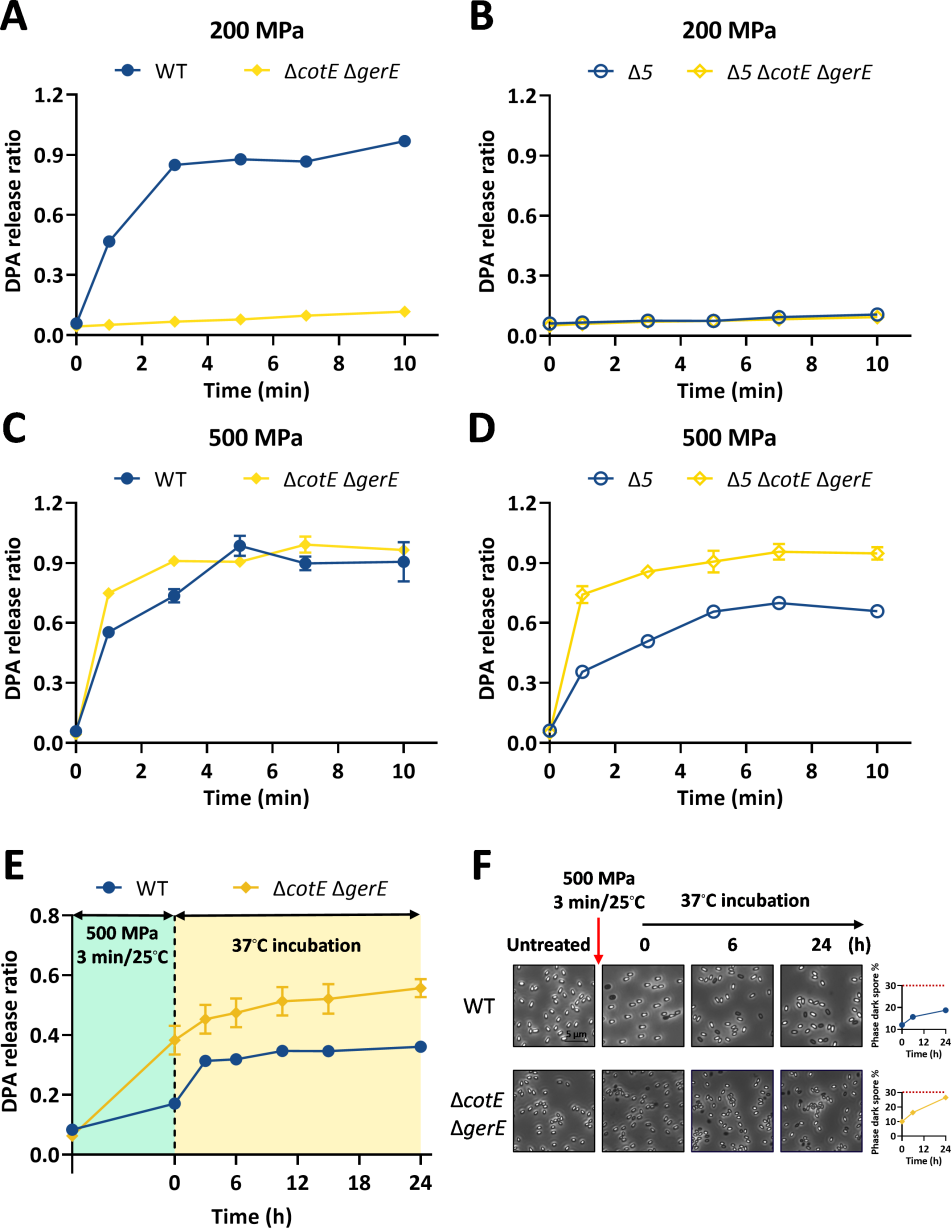
Germination behavior and post-incubation changes of coat-defective spores under HHP treatment. (**A–D**) Germination kinetics of coat-defective spores (Δ*cotE* Δ*gerE* and Δ*5* Δ*cotE* Δ*gerE*) in response to HHP at 200 MPa (**A, B**) or 500 MPa (**C, D**) for 1–10 min at 30°C. Data are presented as the mean ± SD from three independent experiments. (**E**) Spontaneous germination kinetics of coat-defective spores following 500 MPa treatment. WT and Δ*cotE* Δ*gerE* spores were treated at 500 MPa for 3 min at 25°C. The DPA release after HHP treatment was measured immediately and defined as the 0-min time point. After centrifugation to remove this DPA, spores were resuspended to an OD_600_ = 0.5 and incubated at 37°C. Spontaneous germination was monitored by quantifying DPA release at the indicated time points over several hours. (**F**) Microscopic analysis of spontaneous germination. (Left) Representative phase-contrast micrographs of HHP-treated spores during incubation at 37°C. (Right) Germination kinetics were quantified as the percentage of phase-dark spores over time. Spores were prepared as detailed in the Materials and Methods.

To investigate the effect of the coat defect on HHP-induced germination, coat-defective spores (Δ*cotE* Δ*gerE* and Δ*5* Δ*cotE* Δ*gerE*) were treated with 200 or 500 MPa at 30°C for 1–10 min. Under 200 MPa, the germination of Δ*cotE* Δ*gerE* spores was almost completely inhibited (Fig. 2A). Specifically, after 10 min of treatment, DPA release from Δ*cotE* Δ*gerE* spores was only 12% ± 0.2%, compared to 97% ± 0.9% for WT spores (Fig. 2A). Notably, under the same conditions, germination was entirely abolished in both Δ*5* and Δ*5* Δ*cotE* Δ*gerE* spores, which lack GRs (Fig. 2B). These results reinforce the established critical role of GRs in moderate-pressure-induced germination (37, 40) and demonstrate that this process can be suppressed by coat impairment (Fig. 2A and B). In contrast, under 500 MPa treatment, Δ*cotE* Δ*gerE* spores exhibited enhanced germination efficiency compared to WT spores (Fig. 2C). Specifically, after 500 MPa treatment for 3 min, Δ*cotE* Δ*gerE* spores reached 91% ± 1% germination, significantly higher than the 74% ± 3% observed for WT spores (Fig. 2C). This phenotype was more pronounced in Δ*5* Δ*cotE* Δ*gerE* spores compared with Δ*5* spores (Fig. 2D). For instance, after 500 MPa treatment for 3 min, the germination ratio of Δ*5* Δ*cotE* Δ*gerE* was 86% ± 2%, while that of Δ*5* was only 51% ± 3%. These findings confirm that GRs are non-essential for very-high-pressure-induced germination (60), and this process could be facilitated by a coat defect (Fig. 2C and D). Consistent with these genetic results, chemically decoated spores (Fig. S2A and B) also exhibit similar germination behavior under 200 or 500 MPa as described above (Fig. S2C through F).

Having previously observed that SD spores undergo spontaneous germination following 500 MPa treatment during post-incubation (4°C–37°C) (46), we investigated whether the coat defect influences this process. To this end, WT and Δ*cotE* Δ*gerE* spores were treated at 500 MPa and 25°C for 3 min and subsequently incubated at 37°C to monitor spontaneous germination via DPA release and phase-contrast microscopy. Although the spontaneous germination *rate* of Δ*cotE* Δ*gerE* spores was lower than that of WT spores (Fig. 2E), the final *total germination* was significantly higher (*P* < 0.05). After 24 h of incubation, DPA release from Δ*cotE* Δ*gerE* spores reached approximately 56%, compared to 36% for WT spores (Fig. 2E). This was corroborated by phase-contrast microscopy, which showed a higher proportion of phase-dark Δ*cotE* Δ*gerE* spores at the endpoint (Fig. 2F). In summary, while the coat defect reduces the rate of spontaneous germination following 500 MPa treatment, it ultimately results in a greater overall germination yield.

### The germination behavior of cortex-modified spores under HHP

**Fig 3:**
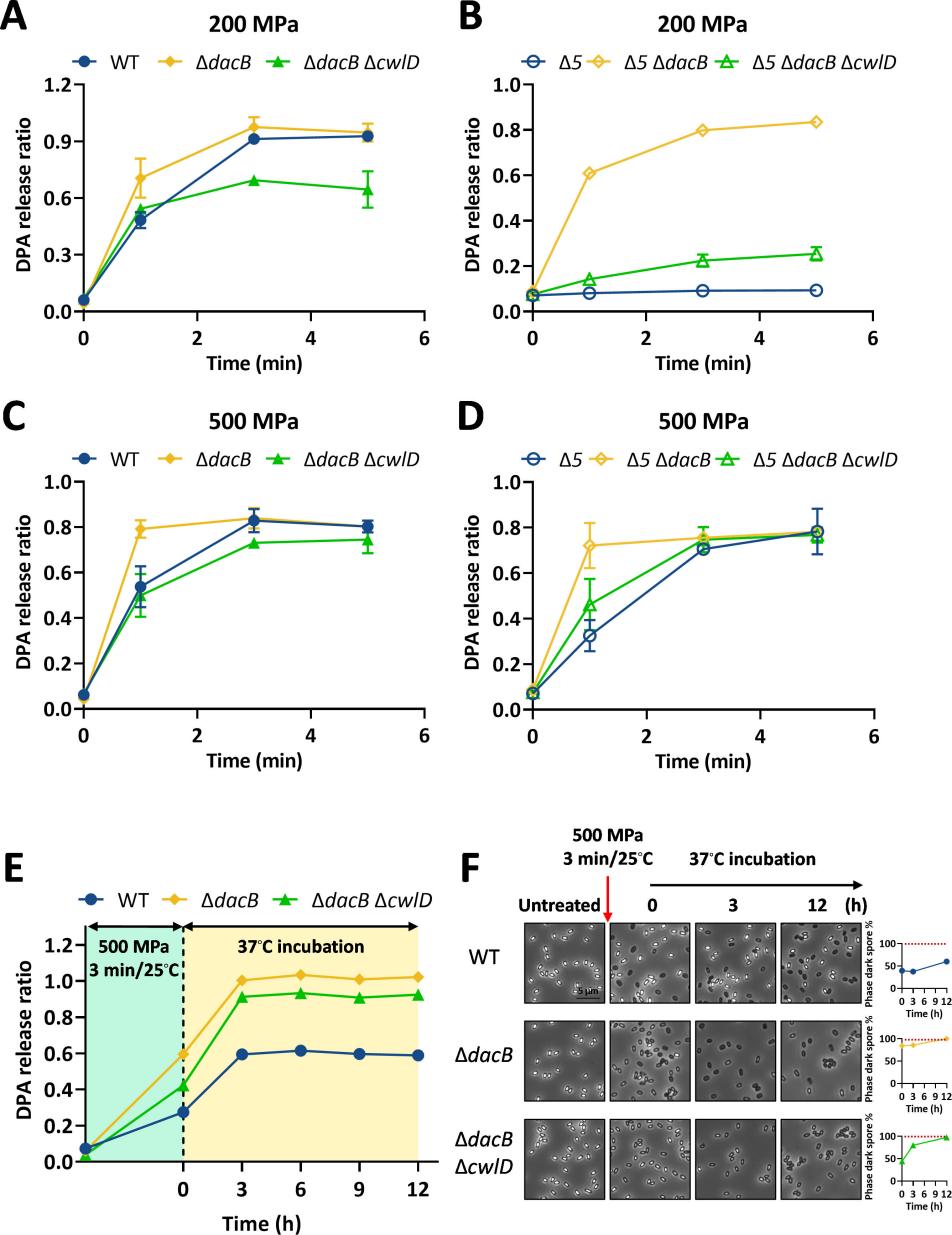
Germination behavior and post-incubation changes of cortex-modified spores under HHP treatment. (**A–D**) Germination kinetics of cortex-modified spores (Δ*dacB*, Δ*dacB* Δ*cwlD*, Δ*5* Δ*dacB,* and Δ*5* Δ*dacB* Δ*cwlD*) in response to HHP at 200 MPa (**A, B**) or 500 MPa (**C, D**) for 1–10 min at 30°C. Data are presented as the mean ± SD from three independent experiments. (**E**) Spontaneous germination kinetics of cortex-modified spores following 500 MPa treatment. WT, Δ*dacB*, and Δ*dacB* Δ*cwlD* spores were treated at 500 MPa for 3 min at 25°C. The DPA release after HHP treatment was measured immediately and defined as the 0-min time point. After centrifugation to remove this DPA, spores were resuspended to an OD_600_ = 0.5 and incubated at 37°C. Spontaneous germination was monitored by quantifying DPA release at the indicated time points over several hours. (**F**) Microscopic analysis of spontaneous germination. (Left) Representative phase-contrast micrographs of HHP-treated spores during incubation at 37°C. (Right) Germination kinetics were quantified as the percentage of phase-dark spores over time. Spores were prepared as detailed in the Materials and Methods.

To investigate the effects of the cortex modification on HHP-induced germination, mutant spores with increased cortex cross-linking (Δ*dacB*, Δ*dacB* Δ*cwlD*, Δ*5* Δ*dacB*, and Δ*5* Δ*dacB* Δ*cwlD*) (Table S1) were treated with 200 or 500 MPa at 30°C for 1–10 min. As shown in Fig. 3A and C, the germination efficiency of Δ*dacB* spores increased under 200 or 500 MPa treatment compared to WT spores (*P* < 0.05). Specifically, after treatment at 30°C for 1 min under 200 or 500 MPa, the DPA release of Δ*dacB* spores was 89% ± 2% and 82% ± 3%, respectively, while that of WT spores was 73% ± 0.3% and 64% ± 2%, respectively. These results indicated that the increased cortex cross-linking exerted a promoting effect on spore germination efficiency under both 200 and 500 MPa. Remarkably, this trend was significantly pronounced in the spores that lacked GRs (Δ*5* Δ*dacB*) (Fig. 3B and D). Especially in Fig. 3B, Δ*5* Δ*dacB* spores without GRs exhibited substantial DPA release under 200 MPa, an unexpected phenotype suggesting that the cortex-crosslinking might have a critical role in modulating HHP-induced spore germination independent of GRs. The complementary expression of DacB in Δ*dacB* mutant spores mitigated the enhanced germination under 200 MPa (Fig. S5A and C), confirming the specific involvement of *dacB* in regulating this process. However, it is notable that this complementation did not fully restore the germination phenotype to wild-type levels, nor did it significantly impact 500 MPa-induced germination (Fig. S5B and D). This implied that, in addition to the loss of *dacB* itself, the deletion of this gene may also affect downstream genes *spmA* and *spmB*, thereby influencing HPP-induced germination (Fig. S5E). Given that the deletion of *spmA* and *spmB* has been reported to increase core water content and induce core expansion (55), we conclude that the increased germination efficiency of Δ*dacB* spores results from the combined effects of increased cortex cross-linking and core expansion. Interestingly, additional deletion of *cwlD* in the Δ*dacB* background decreased the germination efficiency of Δ*dacB* spores (Fig. 3A through D). For instance, after treatment with 200 MPa for 5 min, the DPA release of Δ*cwlD* Δ*dacB* spores was 65% ± 10%, significantly lower than that of Δ*dacB* spores (95% ± 5%, *P* < 0.05) (Fig. 3A). This inhibitory effect likely reflects the role of CwlD in cortex hydrolysis via CLEs, a process essential for efficient DPA release during spore germination (56, 61, 62).

Similarly, to investigate the effect of cortex cross-linking on spontaneous germination, spores were treated at 500 MPa and 25°C for 3 min, followed by immediate incubation at 37°C. During post-incubation, the spontaneous germination rate of Δ*dacB* spores was higher than that of WT spores (Fig. 3E). It was also observed that a further increase in cortex cross-linking (as in Δ*dacB ΔcwlD* spores) led to an additional acceleration in spontaneous germination (Fig. 3E). After 12 h of post-incubation, DPA release reached 102% ± 3% for Δ*dacB* and 93% ± 0.4% for Δ*dacB* Δ*cwlD* spores, both significantly higher than the 59% ± 2% observed for WT spores (*P* < 0.05; Fig. 3E). Phase-contrast microscopy confirmed that nearly all mutant spores had germinated, in contrast to only about 60% of WT spores (Fig. 3F). Together, these data demonstrate that a higher degree of cortex cross-linking enhances both the rate and final extent of spontaneous germination after HHP treatment.

### The germination behavior of the IM-modified spores under HHP

**Fig 4:**
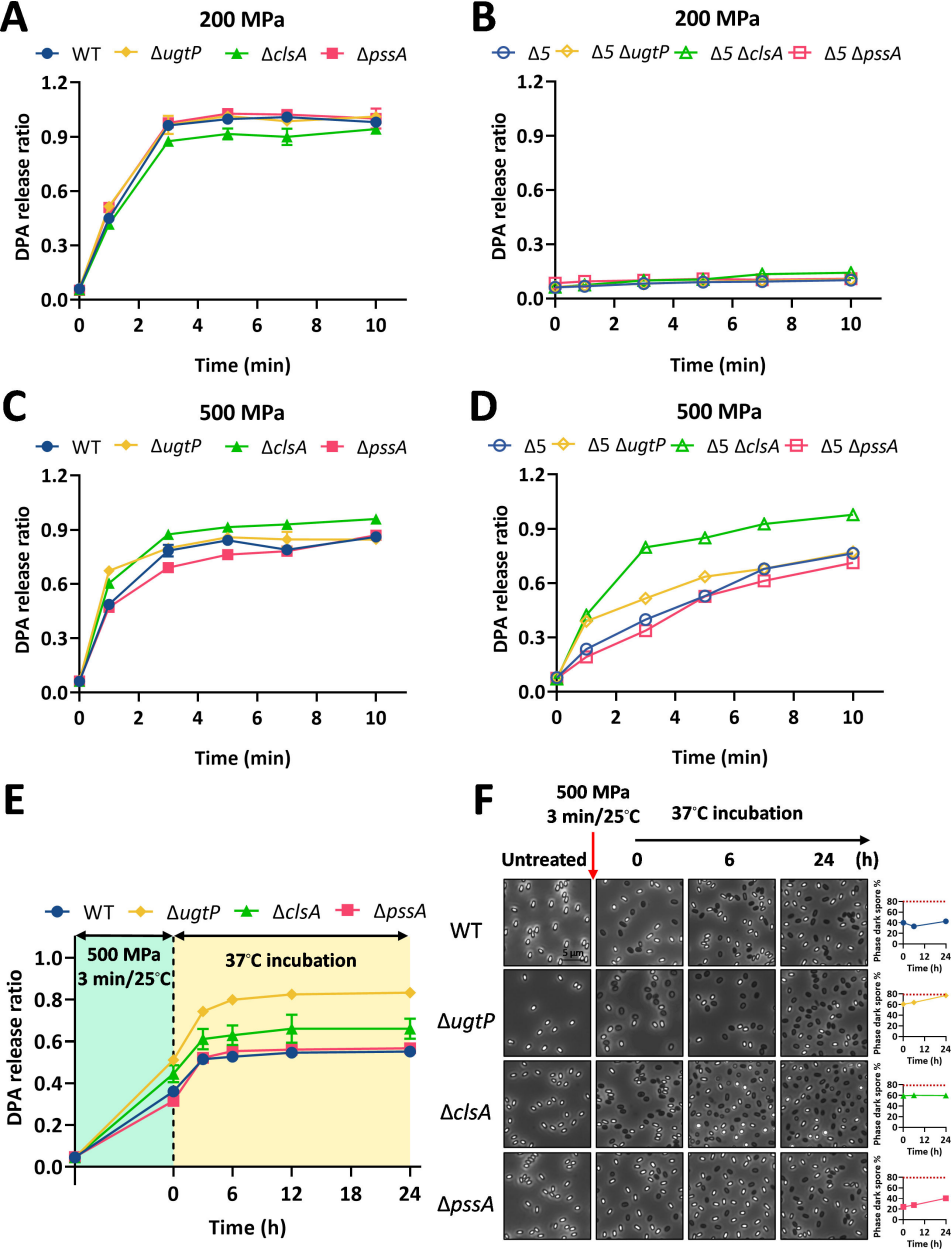
Germination behavior and post-incubation changes of IM lipid-modified spores under HHP treatment. (**A–D**) Germination kinetics of IM lipid-modified spores (Δ*ugtP*, Δ*clsA*, and Δ*pssA*) in response to HHP at 200 MPa (**A, B**) or 500 MPa (**C, D**) for 1–10 min at 30°C. Data are presented as the mean ± SD from three independent experiments. (**E**) Spontaneous germination kinetics of IM lipid-modified spores following 500 MPa treatment. WT, Δ*ugtP*, Δ*clsA,* and Δ*pssA* spores were treated at 500 MPa for 3 min at 25°C. The DPA release after HHP treatment was measured immediately and defined as the 0-min time point. After centrifugation to remove this DPA, spores were resuspended to an OD_600_ = 0.5 and incubated at 37°C. Spontaneous germination was monitored by quantifying DPA release at the indicated time points over several hours. (**F**) Microscopic analysis of spontaneous germination. (Left) Representative phase-contrast micrographs of HHP-treated spores during incubation at 37°C. (Right) Germination kinetics were quantified as the percentage of phase-dark spores over time. Spores were prepared as detailed in the Materials and Methods.

To assess the effect of IM lipid composition on spore germination behavior under HHP, IM mutant spores (Δ*ugtP*, Δ*clsA*, and Δ*pssA*, with, respectively, decreased levels of diglucosyl-1,2-diacylglycerol [dGDG], CL synthase, and phosphatidylethanolamine [PE] of the spore’s IM) (28, 29) (Table S1) were treated with 200 or 500 MPa at 30°C for 1–10 min. As shown in Fig. 4A, the germination efficiency of these IM-modification spores did not change significantly under 200 MPa treatment at 30°C compared to WT spores (*P* > 0.05). No germination was observed in IM-modified spores lacking GRs (Fig. 4B), indicating that these lipid alterations do not directly activate the DPA channel at moderate pressure. In contrast, under 500 MPa, Δ*clsA* spores exhibited enhanced germination compared to WT (Fig. 4C). For instance, after 1 min at 500 MPa, DPA release of Δ*clsA* spores reached 60% ± 0.5%, significantly higher than 49% ± 1% observed in WT spores (Fig. 4C). This trend was more pronounced in the mutant spores lacking GRs (Fig. 4D). Specifically, after treatment at 500 MPa for 3 min, the DPA release of Δ*5* Δ*clsA* spores was approximately twofold higher than WT spores (Fig. 4D). These results suggest that loss of cardiolipin synthase facilitates very-high-pressure-induced germination.

We next evaluated whether changes in IM lipid composition influence spontaneous germination after HHP treatment. Spores were treated at 500 MPa and 25°C for 3 min, then incubated at 37°C. The spontaneous germination rate of Δ*ugtP* spores was significantly higher than that of the WT (*P* < 0.05; Fig. 4E). After 24 h of incubation, the DPA release from Δ*ugtP* spores reached about 83%, a level significantly exceeding that of WT spores (57%; *P* < 0.05). Phase-contrast microscopy confirmed these results, revealing a germination ratio of 78% for Δ*ugtP* spores compared to only 43% for WT spores (Fig. 4F). These results indicate that loss of dGDG (Δ*ugtP*) enhances the spontaneous germination following HHP treatment.

### The germination behavior of DNA protection protein-defective spores under HHP

**Fig 5:**
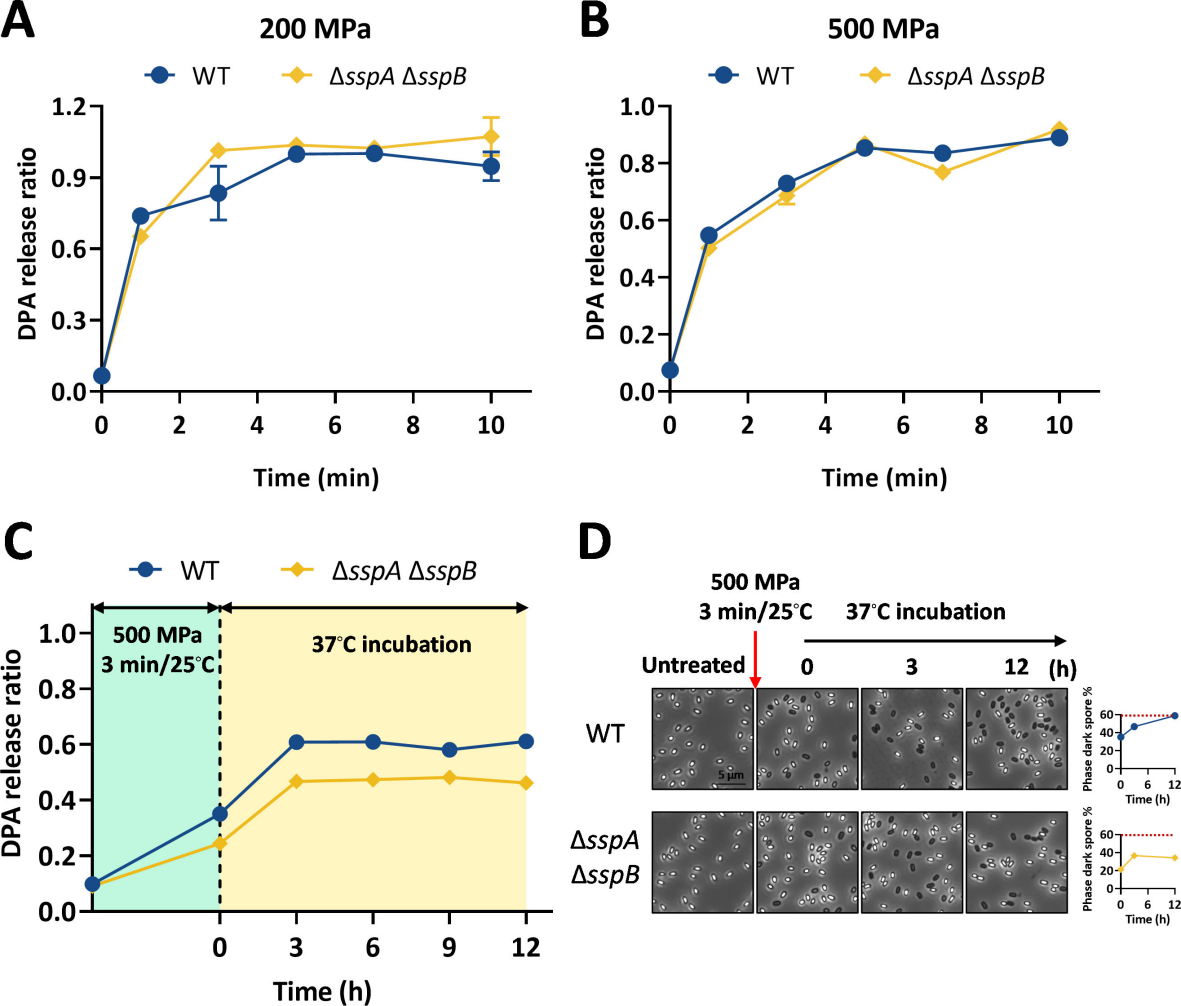
Germination behavior and post-incubation changes of DNA protection proteins-modified spores under HHP treatment. (**A and B**) Germination kinetics of DNA protection proteins-modified spores (Δ*sspA* Δ*sspB*) in response to HHP at 200 MPa (**A**) or 500 MPa (**B**) for 1–10 min at 30°C. Data are presented as the mean ± SD from three independent experiments. (**C**) Spontaneous germination kinetics of DNA protection proteins-modified spores following 500 MPa treatment. WT and Δ*sspA* Δ*sspB* spores were treated at 500 MPa for 3 min at 25°C. The DPA release after HHP treatment was measured immediately and defined as the 0-min time point. After centrifugation to remove this DPA, spores were resuspended to an OD_600_ = 0.5 and incubated at 37°C. Spontaneous germination was monitored by quantifying DPA release at the indicated time points over several hours. (**D**) Microscopic analysis of spontaneous germination. (Left) Representative phase-contrast micrographs of HHP-treated spores during incubation at 37°C. (Right) Germination kinetics were quantified as the percentage of phase-dark spores over time. Spores were prepared as detailed in the Materials and Methods.

To investigate the effect of DNA protection protein deficiency of spores on HHP-induced germination, mutant spores (Δ*sspA* Δ*sspB*) were treated with 200 or 500 MPa at 30°C for 1–10 min. As shown in Fig. 5A and B, following treatment with 200 or 500 MPa, there was no significant difference in germination efficiency between Δ*sspA* Δ*sspB* and WT spores (*P* > 0.05). These results demonstrate that deletion of α/β-type SASPs has no effect on spore germination efficiency under HHP treatment.

To investigate the effect of DNA protection protein deficiency on spontaneous germination, mutant spores were subjected to treatment with 500 MPa at 25°C for 3 min, followed by immediate incubation at 37°C. As shown in Fig. 5C, Δ*sspA* Δ*sspB* spores exhibited lower DPA release than WT spores after HHP treatment (500 MPa/25°C/3 min). In contrast, no significant difference in DPA release was observed between the Δ*sspA* Δ*sspB* spores and WT spores when treated at 500 MPa and 30°C (Fig. 5A). This result suggested a potential temperature-dependent effect on HHP-induced germination for Δ*sspA* Δ*sspB* spores. During post-incubation, the spontaneous germination rate of Δ*sspA* Δ*sspB* spores was similar to that of WT spores, while the total germination ratio was lower than that of WT spores (Fig. 5C). Specifically, the DPA release from Δ*sspA* Δ*sspB* spores was 46% ± 0.8%, which was significantly lower than that of the WT (61% ± 0.6%, *P* < 0.05; Fig. 5C). Phase contrast microscopy observations confirmed the same trend. These results indicate that the DNA protection protein modifications do not influence spontaneous germination rate, while slightly decreasing the final germination yield depending on the HHP treatment temperature.

### The effects of structural modifications on spore resistance to HHP

**Fig 6:**
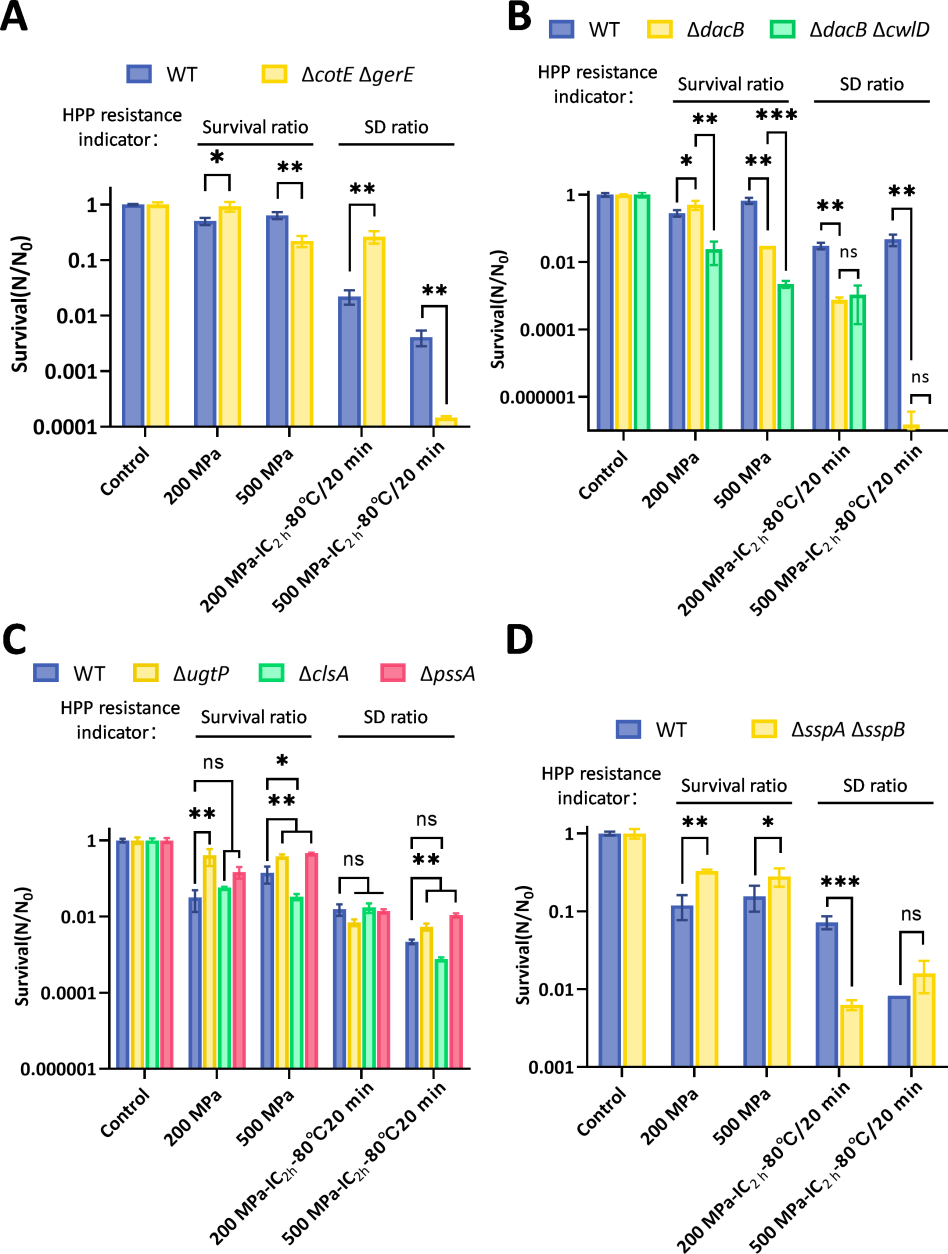
Survival and superdormancy of structurally modified *B. subtilis* spores after HHP treatment. Analysis of survival and superdormancy ratios in mutant spores after treatment at 200 or 500 MPa (10 min, 37°C). The strains evaluated are (**A**) coat-defective (Δ*cotE* Δ*gerE*), (**B**) cortex-modified (Δ*dacB*, Δ*dacB* Δ*cwlD*), (**C**) inner membrane lipid-modified (Δ*ugtP*, Δ*clsA*, Δ*pssA*), and (**D**) DNA protection-deficient (Δ*sspA* Δ*sspB*). The SD spore population was determined by assessing viability after a 2 h incubation at 37°C and subsequent pasteurization (80°C, 20 min). Data are presented as the mean ± SD from three independent experiments. *P < 0.05, **P < 0.01, ***P < 0.001 and ns, no significant difference.

To investigate the effects of spore intrinsic structures on HHP resistance, spores with modifications in the coat, cortex, IM, and DNA protection proteins were treated with 200 or 500 MPa at 30°C for 10 min. Since HHP alone has limited direct inactivation capacity for spores, effective spore inactivation relies on a “germination-inactivation” strategy (36, 63). Given that the proportion of SD spores is a critical determinant of the effectiveness of this approach, we assessed spore resistance to HHP based on both survival ratio (direct indicators) and SD ratio (indirect indicators) of spores following HHP treatment (Fig. 6). Specifically, the HHP-treated spores were either directly tested for survival ratio or subsequently subjected to incubation (37°C/2 h) (IC_2 h_), followed by heat treatment (80°C/20 min) for examining the SD ratio.

As shown in Fig. 6A, after 200 MPa treatment, coat-defective (Δ*cotE* Δ*gerE*) spores exhibited higher survival and SD ratios than WT spores, indicating increased resistance at this pressure. In contrast, after 500 MPa, both survival and SD ratios of Δ*cotE* Δ*gerE* spores were significantly lower than those of WT spores (*P* < 0.05). Thus, a coat defect enhances resistance at 200 MPa but reduces it at 500 MPa. This inverse relationship between germination efficiency (Fig. 2A and C) and survival/SD ratios (Fig. 6A) highlights the coat’s role in modulating pressure-dependent resistance.

As shown in Fig. 6B, under 200 MPa treatment, spores with increased cortex cross-linking (Δ*dacB*) showed a slightly elevated survival rate but a significantly reduced SD ratio compared to WT spores (Fig. 6B), suggesting that enhanced cross-linking lowers resistance at 200 MPa primarily by decreasing the SD subpopulation. Under 500 MPa treatment, both survival and SD ratios of Δ*dacB* spores were significantly lower than those of WT spores (*P* < 0.05). Additional deletion of *cwlD* in the Δ*dacB* background further reduced survival under both pressures (*P* < 0.05) without affecting the SD ratio. These data strongly indicate that increased cortex cross-linking generally decreases spore HHP resistance.

As shown in Fig. 6C, under 500 MPa treatment, Δ*clsA* spores (lacking cardiolipin synthase) showed significantly lower survival than WT spores (*P* < 0.05), with a numerically lower SD ratio (*P* > 0.05). This indicates that cardiolipin deficiency reduces resistance to 500 MPa. The other IM mutant spores did not exhibit a significantly reduced resistance at 200 or 500 MPa. For example, under 200 MPa treatment, the survival ratios of Δ*ugtP* spores were slightly increased, but the SD ratio showed no difference (*P* > 0.05). Under 500 MPa treatment, the survival and SD ratios of Δ*ugtP* and Δ*pssA* spores were slightly increased (*P* < 0.05).

As shown in Fig. 6D, after 200 MPa treatment, Δ*sspA* Δ*sspB* spores had a slightly higher survival ratio but a significantly lower SD ratio than WT spores (*P* < 0.05). Under 500 MPa treatment, the survival ratio of Δ*sspA* Δ*sspB* spores was slightly higher than WT spores, while the SD ratio showed no difference (*P* > 0.05). These results indicate that the deficiency of α/β-type SASPs reduces the resistance of spores at 200 MPa via decreasing the proportion of SD spores, while showing minimal effect at 500 MPa.

## DISCUSSION

In this study, we systematically evaluated how structural modifications in key spore components, namely the coat, cortex, IM, and DNA-protective proteins, influence germination and inactivation under HHP. The principal findings can be summarized as follows: (i) spore coat defects diminished germination efficiency at 200 MPa yet enhanced it at 500 MPa (Fig. 2A through D). This differential response led to an enlarged SD subpopulation at the lower pressure and a reduced SD ratio at the higher pressure, ultimately increasing spore resistance at 200 MPa but decreasing it at 500 MPa (Fig. 6A). (ii) Increased cortex cross-linking significantly promoted germination efficiency under both 200 and 500 MPa (Fig. 3A through D). The consequent reduction in the SD ratio under both pressure conditions resulted in decreased spore resistance (Fig. 6B). (iii) Reduced CL content in the IM led to higher germination efficiency, specifically at 500 MPa (Fig. 4C and D). This increase contributed to a lower SD ratio and thus reduced spore resistance at this pressure level (Fig. 6C). (iv) Absence of α/β-type SASPs did not significantly affect germination efficiency or overall resistance at 500 MPa. However, at 200 MPa, it reduced spore resistance by lowering the SD ratio (Fig. 5A, B and 6D).

**Fig 7:**
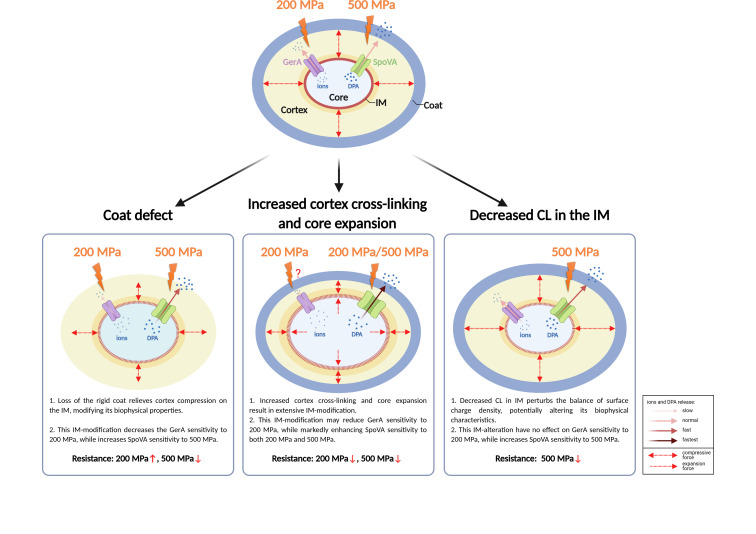
Model depicting the effect of structural modification of *Bacillus subtilis* spores on their resistance to HHP. The model elucidates how coat defect, increased cortex cross-linking, or decreased cardiolipin (CL) in the inner membrane (IM) alters the biophysical properties of IM, thereby differentially modulating the sensitivity of GerA and SpoVA channels to HHP and ultimately shaping spore HHP resistance.

The first major finding of this study was that the spore coat defect (Δ*cotE* Δ*gerE*) reduced germination efficiency at 200 MPa but enhanced it at 500 MPa. Although the role of coat defects in HHP-induced germination has not been previously investigated, their impact on nutrient-induced germination is well documented. For instance, coat deficiencies have been shown to impair nutrient-induced germination due to the loss of coat-localized GerP channels, which facilitate nutrients (L-alanine or AGFK) access to the GerA germinant receptor (Fig. S3A through D) (64). In contrast, the enhanced DDA-induced germination efficiency caused by coat defect is likely attributed to alterations in IM properties, as DDA is proposed to primarily act on the IM to open the DPA channel and trigger germination (Fig. S3E and F) (65). Coat defects also increase core water content and reduce heat resistance of spores (22), suggesting underlying changes in IM organization and permeability. These observations can be interpreted within a structural framework (Fig. 7): under normal conditions, the expanded cortex peptidoglycan layer compresses the core, limiting its hydration (66, 67), while the rigid coat constrains the cortex and transmits pressure to the IM (30, 56). Loss of the coat may alleviate this constraint, reducing compressive force on the IM and thereby altering its biophysical properties (10). Such structural modifications could subsequently affect the function of key germination proteins—GRs and SpoVA channels—resident in the IM. HHP is known to induce germination through pressure-dependent mechanisms: at 50–300 MPa, it primarily activates GRs (GerA, GerB, GerK), whereas above 400 MPa, it opens SpoVA channels to induce DPA release (37, 40). In both cases, HHP may act directly on the proteins themselves or indirectly through effects on the surrounding IM (38, 68). Therefore, it is plausible that coat defect-induced alterations in the IM somehow inhibit GR-mediated germination at 200 MPa while facilitating SpoVA-dependent germination at 500 MPa (Fig. 7). The precise mechanism underlying this differential response warrants further investigation.

The second major finding of this work is that increased cortex cross-linking, as well as core expansion (resulting from the Δ*dacB* mutation), significantly enhanced spore germination efficiency under both 200 and 500 MPa HHP treatments. The cortex is known to maintain core dehydration by exerting an expansion-driven compressive force (66, 67), which is largely determined by the extent of cortex cross-linking (56). The *dacB* mutation increases cross-linking of the cortex (55), potentially reducing the expansion effect and thus could reasonably relieve the compressive force on the IM. Meanwhile, the impaired expression of the downstream genes *spmA* and *spmB* induced by the *dacB* mutation likely results in increased core water content and core expansion (55), thereby potentially expanding the IM. These effects could significantly alter IM biophysical properties and the functional state of embedded germination proteins—GRs and SpoVA channels—thereby influencing germination. Supporting this view, Δ*dacB* spores exhibited accelerated germination in response to DDA, an agent that acts directly on the IM (Fig. S4E and F) (62). Thus, the enhanced HHP-induced germination in Δ*dacB* spores likely results from changes in IM properties or germination protein activity mediated by cortex cross-linking and core expansion (Fig. 7). A particularly revealing observation was that even in Δ5 Δ*dacB* spores without GRs, substantial DPA release occurred at 200 MPa (Fig. 3B)—a condition under which GRs are typically essential for germination initiation (40). This result provides compelling evidence that increased cortex cross-linking and core expansion render the SpoVA channel highly susceptible to direct activation by HHP (Fig. 7), bypassing the requirement for GR signaling even at moderate pressures such as 200 MPa.

As discussed above, coat defects, along with increased cortex cross-linking and core expansion, are likely to alter the physical properties of the IM and the functional state of associated germination proteins (Fig. 7). These structural modifications similarly enhanced germination efficiency under 500 MPa, as evidenced by the promoted germination in both mutant strains (Fig. 2C, D, 3C and D). In contrast, under 200 MPa, the two perturbations led to divergent germination behaviors: coat deficiency impaired germination (Fig. 2A and B), whereas elevated cortex cross-linking and core expansion facilitated it (Fig. 3A and B). A plausible explanation is that both alterations inhibit GerA-mediated germination (Fig. S3A and S4A) while simultaneously promoting the opening of SpoVA channels (Fig. S3E and S4E). In the case of the highly cross-linked cortex and expanded core, however, the SpoVA complex appears to become exceptionally susceptible to activation—even at moderate pressure—thereby overriding the inhibitory effect on GerA function (Fig. 7). This interpretation is strongly supported by the substantial DPA release observed at 200 MPa in GR-deficient Δ*5* Δ*dacB* spores (Fig. 3B), which would otherwise remain dormant under such conditions. Nevertheless, the precise biophysical mechanisms through which coat integrity, cortex architecture, and core expansion modulate IM properties or germination protein activity remain unclear. Elucidating these aspects will be essential for a comprehensive understanding of HHP-induced spore germination.

The third finding of this study is that a reduction in CL content within the IM enhanced spore germination efficiency under 500 MPa (Fig. 4C). CL represents a major anionic phospholipid in the spore IM, and its deficiency has previously been associated with elevated core water content (29). As the negative charge conferred by CL is critical for maintaining membrane potential, ion gradients, and the functional integrity of membrane‐embedded proteins (69), its absence may disrupt the balance of surface charge density. This alteration could, in turn, affect the activity or pressure sensitivity of protein complexes, such as the SpoVA‐encoded DPA channel, thereby modulating germination responsiveness under very high pressure (Fig. 7). Nevertheless, the precise mechanisms through which sporulation‐associated changes in IM lipid composition influence the structural and functional states of membrane‐localized germination machinery remain to be fully elucidated.

Our study also finds that the absence of DNA-protecting proteins, specifically α/β-type SASPs, reduced the proportion of SD spores following treatment at 200 MPa. HHP is thought to exert its sterilizing effect primarily through damage to membrane lipids and proteins, with DNA damage occurring largely as a secondary consequence (70). Consistent with this view, the deletion of SASP-encoding genes (Δ*sspA* Δ*sspB*) had only a marginal effect on HHP-induced germination efficiency (Fig. 5A and B). However, the SD spore fraction recovered after 200 MPa treatment was significantly lower in the SASP-deficient mutant than in the wild type (Fig. 6D). Previous work has established that SASPs help maintain spore dormancy, and their absence increases the propensity for spontaneous germination (71). Our results align with this mechanism: although Δ*sspA* Δ*sspB* spores germinated at a rate similar to wild-type spores under immediate HHP stress at 200 MPa, the population that remained dormant was more prone to initiate germination in response to subsequent environmental cues, such as incubation conditions and mild heat, during the post-treatment assessment. This heightened responsiveness ultimately led to a smaller residual SD subpopulation.

### Conclusion

In this study, we systematically elucidated the distinct contributions of key spore structural components—the coat, cortex, IM, and DNA protection machinery—to HHP-induced germination and inactivation, which collectively define spore HHP resistance. Coat defects altered the balance between GerA-mediated germination and SpoVA channel activation under pressure, resulting in increased resistance at 200 MPa but decreased resistance at 500 MPa. Enhanced cortex cross-linking and core expansion significantly promoted SpoVA channel opening even at moderate pressure, thereby reducing spore resistance across both 200 and 500 MPa conditions. Similarly, a reduction in CL content in the IM likely impaired membrane charge regulation, facilitating germination and diminishing resistance specifically at 500 MPa. Together, these results demonstrate that multiple structural elements confer HHP resistance through diverse mechanisms, with structural modifications that influence IM biophysical properties or the function of germination proteins—such as GRs and SpoVA channels—serving as critical regulators of pressure-induced germination. Further investigation is needed to precisely delineate how structural changes remodel the IM and modulate the activity of germination-associated proteins under high pressure.

## SUPPLEMENTAL MATERIAL

Supplemental material aem.00070-26-s0001.pdf Fig. S1 to S5; Tables S1 and S2.

ASM does not own the copyrights to Supplemental Material that may be linked to, or accessed through, an article. The authors have granted ASM a non-exclusive, world-wide license to publish the Supplemental Material files. Please contact the corresponding author directly for reuse.
