# Supplementary material for: New insights into the structure-based mechanism of Bacillus subtilis spore resistance to high hydrostatic pressure
Source: Appl Environ Microbiol. 2026 Apr 27;92(5):e00070-26. doi: 10.1128/aem.00070-26 (PMC13188902; doi:10.1128/aem.00070-26)
Supplement: Supplemental material — Fig. S1 to S5; Tables S1 and S2. [file aem.00070-26-s0001.pdf]

Supplementary Figure Legends

Figure S1. Effects of structural modifications on spore resistance.

(A) NaClO resistance assay: Δ*5* and coat-defective spores (Δ*5* Δ*cotE* Δ*gerE*) were exposed to 2.5% (0.34 M) NaClO at 23 °C 5 min, and the spores were neutralized with 0.1 M sodium thiosulfate. **(B-C)** Heat resistance assay: **(B)** Δ*5* and cortex-modified spores (Δ*5* Δ*dacB*, Δ*5* Δ*dacB* Δ*cwlD*); **(C)** Δ*5* and IM-modified spores (Δ*5* Δ*ugtP*, Δ*5* Δ*clsA*, and Δ*5* Δ*pssA*) were treated at 93 °C for 10 min. All experiments were performed in triplicate, and data are presented as the mean ± SD of three independent replicates.

Figure S2. Assessment of coat integrity and DPA release in PY- and Δ*5*-decoated spores under 200 MPa or 500 MPa treatment.

(A) Lysozyme sensitivity assay. The germination of intact (PY, Δ*5*) and decoated spores (PY-decoated, Δ*5*-decoated) was monitored by measuring the decrease in OD_{600} over time upon exposure to 25 mg/mL lysozyme. **(B)** Phase-contrast micrographs of spores before and after treatment with 25 mg/mL lysozyme for 20 min. **(C-F)** Dipicolinic acid (DPA) release kinetics of PY, PY-decoated (C, E) and Δ*5*, Δ*5*-decoated (D, F) spores under 200 MPa (C, D) or 500 MPa (E, F). Data are presented as the mean ± SD from three independent experiments.

Figure S3. Germination kinetics of coat-defective spores induced by nutrient (L-alanine, AGFK) and non-nutrient (DDA) germinants.

Coat-defective spores (Δ*cotE* Δ*gerE* and Δ*5* Δ*cotE* Δ*gerE*) were heat-activated and germinated by **(A, B)** 10 mM L-alanine, **(C, D)** AGFK (10 mM L-asparagine, 5 mg/mL D-glucose, 5 mg/mL D-fructose, 50 mM KCl), or **(E, F)** 1 mM dodecylamine (DDA). Germination was monitored by measuring the DPA release over time. Data are presented as the mean ± SD from three independent experiments.

Figure S4. Germination kinetics of cortex-modified spores induced by nutrient (L-alanine, AGFK) and non-nutrient (DDA) germinants.

Cortex-modified spores (Δ*dacB*, Δ*dacB* Δ*cwlD,* Δ*5* Δ*dacB*, and Δ*5* Δ*dacB* Δ*cwlD*) were heat-activated and germinated by **(A, B)** 10 mM L-alanine, **(C, D)** AGFK (10 mM L-asparagine, 5 mg/mL D-glucose, 5 mg/mL D-fructose, 50 mM KCl), or **(E, F)** 1 mM DDA. Germination was monitored by measuring the DPA release over time. Data are presented as the mean ± SD from three independent experiments.

Figure S5. Germination behavior of the *dacB*-complemented strain under HHP treatment and

sporulation efficiency of *spmA spmB* mutants.

(A-D) Germination kinetics of *dacB*-complemented strain spores (△*dacB dacB*, △*5* Δ*dacB dacB*) in response to HHP at 200 MPa **(A, C)** or 500 MPa **(B, D)** for 1-10 min at 30°C. **(E)** The sporulation efficiency of mutants strains (Δ*dacB* and Δ*spmA spmB*) was calculated as [(the CFU count after heat treatment at 80°C for 20 min) / (the CFU count without heat treatment)] × 100%. Samples were collected at the late sporulation phase (37°C and 22 hours culturing in 20 ml liquid DSM medium) for this assay.

Figure S1

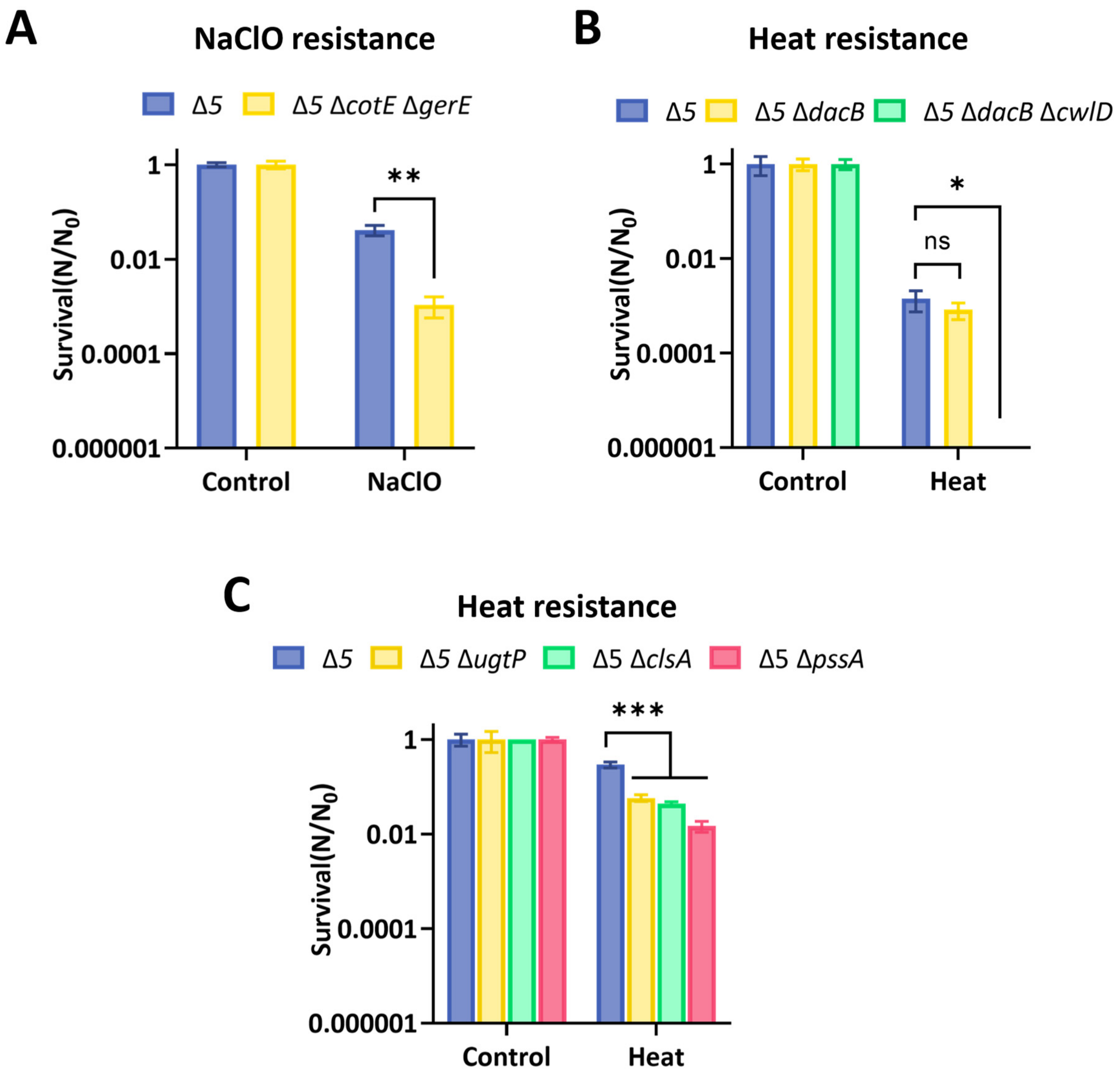

A
NaClO resistance
Δ5
Δ5 ΔcotE ΔgerE
Survival(N/N0)
1
0.01
0.0001
0.000001
**
Control
NaClO
B
Heat resistance
Δ5
Δ5 ΔdacB
Δ5 ΔdacB ΔcwlD
Survival(N/N0)
1
0.01
0.0001
0.000001
*
ns
Control
Heat
C
Heat resistance
Δ5
Δ5 ΔugtP
Δ5 ΔclsA
Δ5 ΔpssA
Survival(N/N0)
1
0.01
0.0001
0.000001
***
Control
Heat

44 **Figure S2**

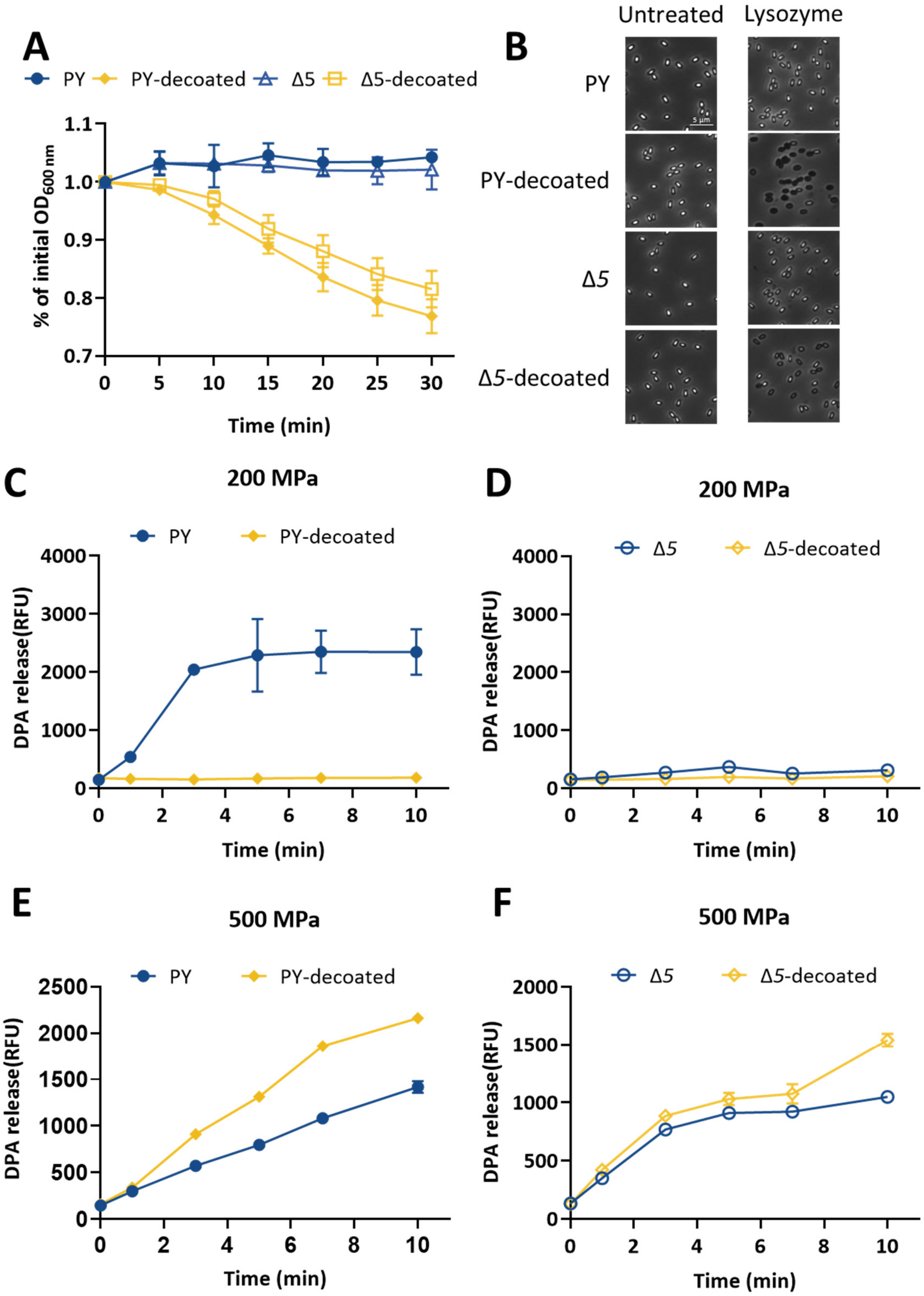

45
46

Figure S3

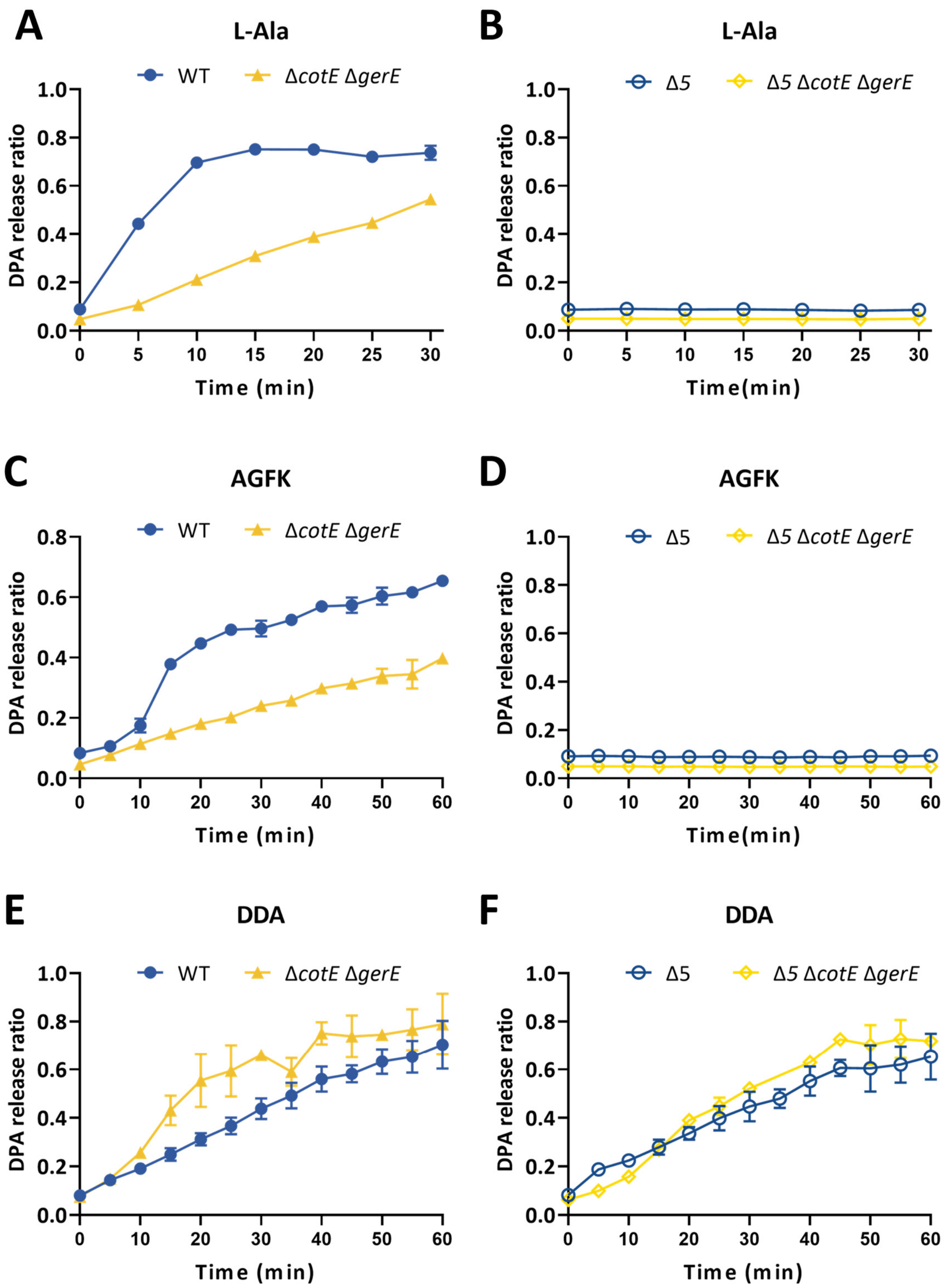

A
L-Ala
WT
ΔcotE ΔgerE
DPA release ratio
1.0
0.8
0.6
0.4
0.2
0.0
0
5
10
15
20
25
30
Time (min)
B
L-Ala
Δ5
Δ5 ΔcotE ΔgerE
DPA release ratio
Time(min)
C
AGFK
WT
ΔcotE ΔgerE
DPA release ratio
Time (min)
D
AGFK
Δ5
Δ5 ΔcotE ΔgerE
DPA release ratio
Time(min)
E
DDA
WT
ΔcotE ΔgerE
DPA release ratio
Time (min)
F
DDA
Δ5
Δ5 ΔcotE ΔgerE
DPA release ratio
Time (min)

Figure S4

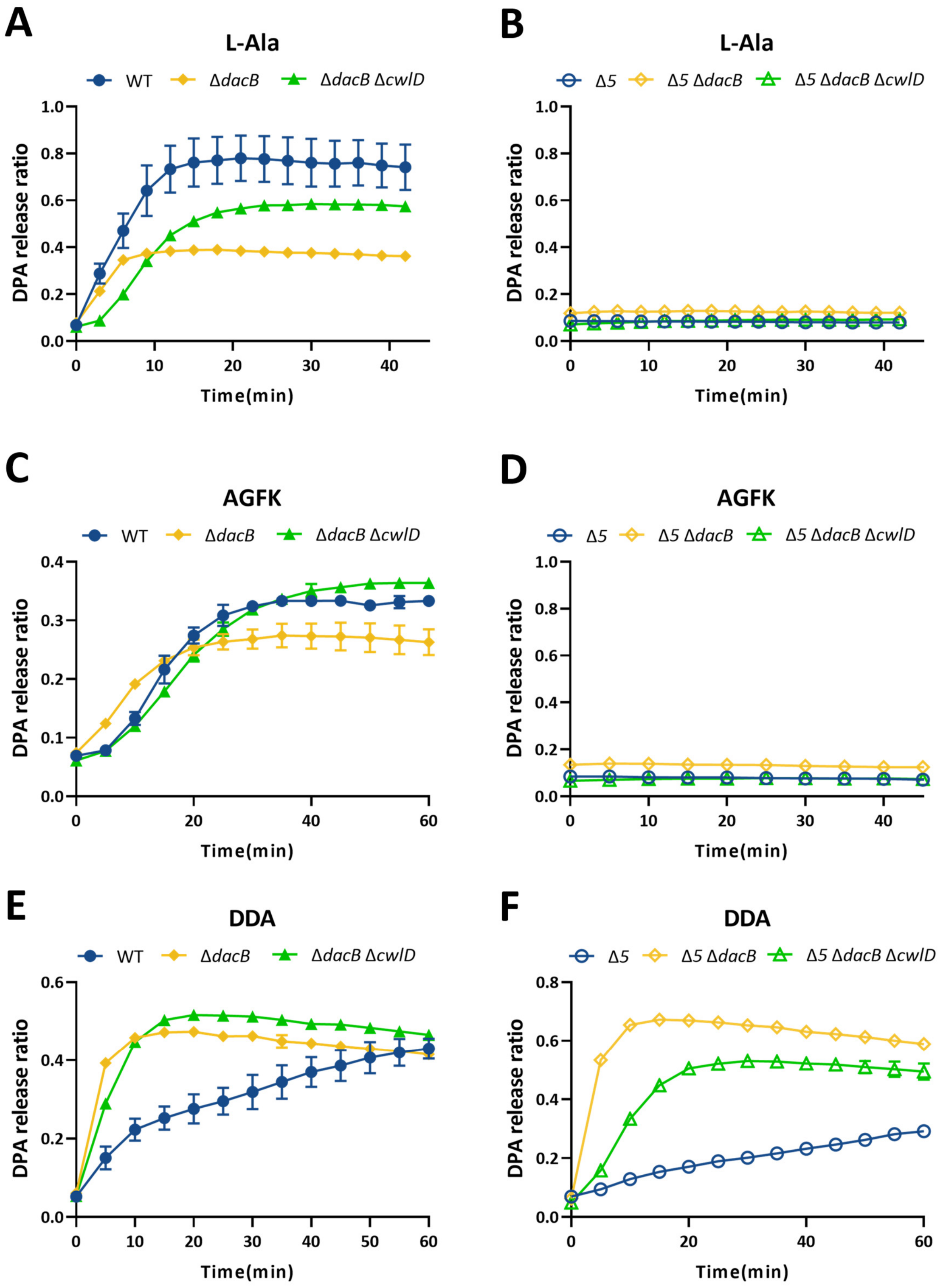
A
L-Ala
WT
ΔdacB
ΔdacB ΔcwlD
DPA release ratio
Time(min)
B
L-Ala
Δ5
Δ5 ΔdacB
Δ5 ΔdacB ΔcwlD
DPA release ratio
Time(min)
C
AGFK
WT
ΔdacB
ΔdacB ΔcwlD
DPA release ratio
Time(min)
D
AGFK
Δ5
Δ5 ΔdacB
Δ5 ΔdacB ΔcwlD
DPA release ratio
Time(min)
E
DDA
WT
ΔdacB
ΔdacB ΔcwlD
DPA release ratio
Time(min)
F
DDA
Δ5
Δ5 ΔdacB
Δ5 ΔdacB ΔcwlD
DPA release ratio
Time(min)

Figure S5

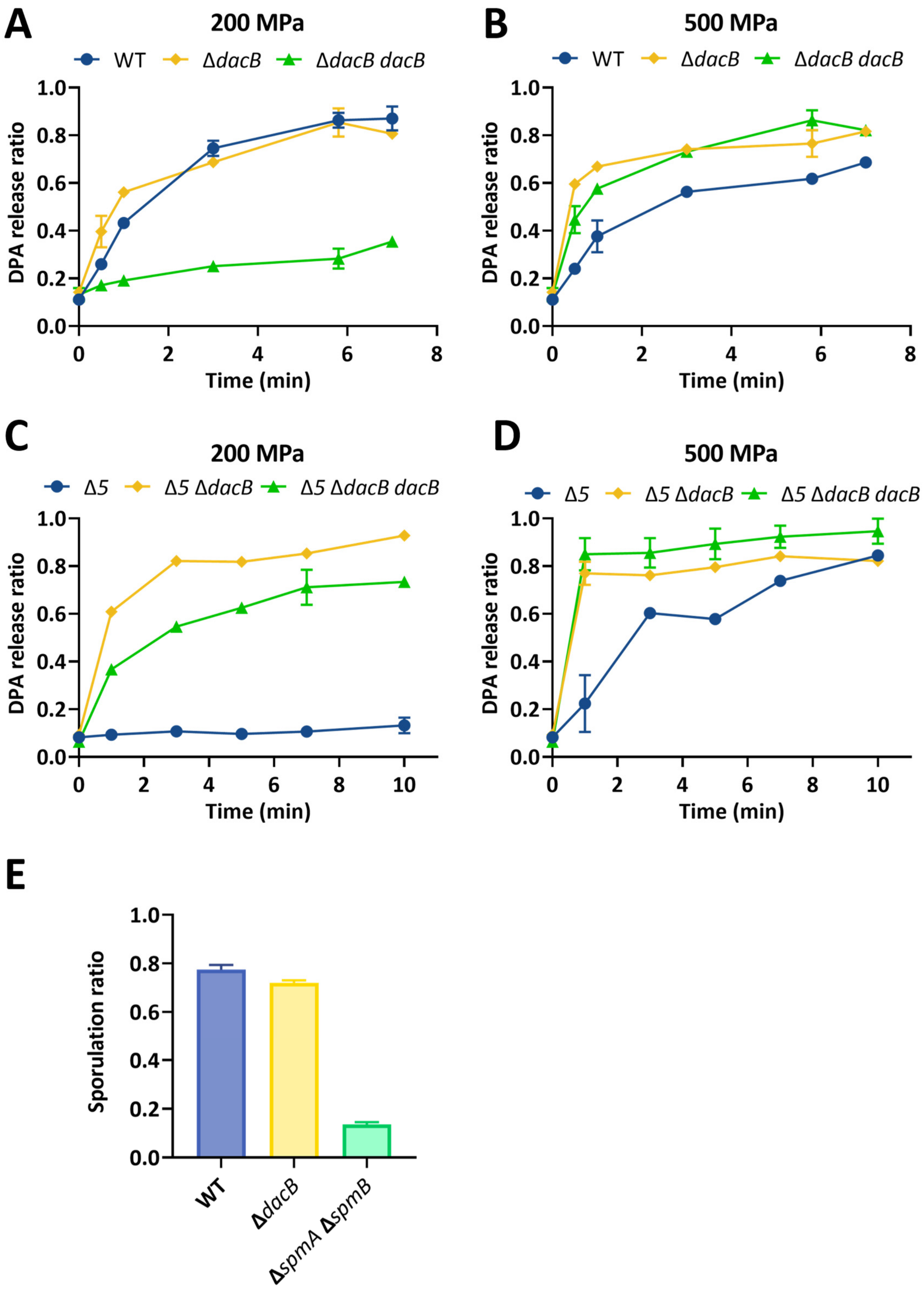
A
200 MPa
WT
ΔdacB
ΔdacB dacB
DPA release ratio
Time (min)
B
500 MPa
WT
ΔdacB
ΔdacB dacB
DPA release ratio
Time (min)
C
200 MPa
Δ5
Δ5 ΔdacB
Δ5 ΔdacB dacB
DPA release ratio
Time (min)
D
500 MPa
Δ5
Δ5 ΔdacB
Δ5 ΔdacB dacB
DPA release ratio
Time (min)
E
Sporulation ratio
WT
ΔdacB
ΔspmA ΔspmB

Table S1. Strains used in this study.

Strain	Genotype	Phenotype	Source	Reference
PS832	Wild type (WT)	*B. subtilis* 168 WT	Lab stock Gifted by Prof. Peter Setlow (UConn Health, US)	(Perkins et al., 2005)
bLA201	*Δ5*	Lacking all GerA-type GRs (Δ*gerBB*, Δ*gerKB*, Δ*yfkT*, Δ*yndE*, Δ*gerA*)	Gifted by Prof. David Rudner (Harvard Medical School)	(Amon et al., 2022)
SNK8123	Δ*cotE::tet* Δ*gerE::kan*	Spores lacking visible coats and decreasing resistance to NaClO.	This work	(Ghosh et al., 2008)
SNK8124	*Δ5* Δ*cotE::tet* Δ*gerE::kan*	Lacking all GerA-type GRs and visible coats.	This work	(Ghosh et al., 2008)
SNK 407	Δ*dacB::kan*	Increasing the degree of cross-linking by 3-4-fold and decreasing resistance to heat.	This work	(Popham et al., 1995)
SNK8126	*Δ5* Δ*dacB::kan*	Lacking all GerA-type GRs and having a 3-4-fold degree of cortex cross-linking.	This work	(Popham et al., 1995)
SNK8125	Δ*dacB* Δ*cwlD::spec*	Spores with an increased cortex cross-linking degree by 9-fold and exhibit an inability of CLEs to recognize and degrade the cortex.		(Popham et al., 1999)
SNK8127	*Δ5* Δ*dacB* Δ*cwlD::spec*	Lacking all GerA-type GRs, have a 9-fold degree of cortex cross-linking and exhibit an inability of CLEs to recognize and degrade the cortex.	This work	(Popham et al., 1999)
SNK1171	Δ*pssA::kan*	Decreasing the level of Phosphatidylethanolamine (PE) of spores' inner membrane (IM).	This work	(Griffiths and Setlow, 2009)
SNK2194	*Δ5* Δ*pssA::kan*	Lacking all GerA-type GRs and decreasing the level of PE of the spores' IM.	This work	(Griffiths and Setlow, 2009)
SNK321	Δ*ugtP::spec*	Decreasing the level of Diglucosyl-1,2-diacylglycerol (dGDG) of spores' IM.	This work	(Griffiths & Setlow, 2009)
SNK325	*Δ5* Δ*ugtP::spec*	Lacking all GRs and decreasing the level of dGDG of spores' IM.	This work	(Griffiths & Setlow, 2009)
SNK1172	Δ*clsA::kan*	Decreasing the level of Cardiolipin (CL) of the spores' IM.	This work	(Griffiths & Setlow, 2009)
SNK2193	*Δ5* Δ*clsA::kan*	Lacking all GerA-type GRs and decreasing the level of CL of the spores' IM.	This work	(Griffiths & Setlow, 2009)
PS533	WT (pUB110)	PS832 carries plasmid pUB110, which provides kanamycin resistance.	Lab stock Gifted by Prof. Peter Setlow (UConn Health, US)	
PS578	Δ*sspA* Δ*sspB::kan*	Lacking α/β-type SASPs ($\alpha^-\beta^-$) and decreasing resistance to radiation.	Lab stock Gifted by Prof. Peter Setlow (UConn Health, US)	(Setlow, 2007; Setlow & Christie, 2021)
PS3328	Δ*cotE::tet*	Lacking much of the spore coat.	Lab stock Gifted by Prof. Peter Setlow (UConn Health, US)	(Schottroff et al., 2019)
BKK28410	Δ*gerE::kan*	Lacking much of the spore coat.	Lab stock	(Ghosh et al., 2008)
BKK23190	Δ*dacB::kan*	Increasing the degree of cortex cross-linking by 3-4-fold and decreasing resistance to	Lab stock	(Popham et al., 1995)

		heat.		
LR48	Δ*cwlD::spec*	Laking muramic-δ-lactam of PG and increasing the degree of cross-linking by 2-fold.	Lab stock	(Popham et al., 1999)
BKK02270	Δ*pssA::kan*	Decreasing the level of PE of spores' IM.	Lab stock	(Griffiths and Setlow, 2009)
BKK36590	Δ*clsA::kan*	Decreasing the level of CL of the spores' IM.	Lab stock	(Griffiths & Setlow, 2009)
ZQ52	Δ*dacB::kan, amyE::PdacB-dacB-cm*	The Δ*dacB* mutant phenotype was complemented by reintroducing a wild-type copy of *dacB* at the amyE locus.	This work	(Popham et al., 1995)
ZQ62	Δ*5*, Δ*dacB::kan, amyE::PdacB-dacB-cm*	Lacking all GerA-type GRs and the Δ*dacB* mutant phenotype was complemented by reintroducing a wild-type copy of *dacB* at the amyE locus.	This work	(Popham et al., 1995)
ZQ63	Δ*spmA spmB::mls*	Lacking *spmA* and *spmB.*	This work	(Popham et al., 1995)

Detailed description of strain construction

The *B. subtilis* strains used in this study are listed in Table S1. Two mutagenesis strategies were employed. In the first strategy, specific primer pairs were designed to amplify the genomic regions flanking the target gene. The resulting PCR products were assembled with an appropriate antibiotic resistance gene using the Gibson Assembly method (NEB, USA), following the protocol outlined by Guérout-Fleury et al. (Guérout-Fleury et al., 1996). The assembly product was then used to transform the *B. subtilis* strain, generating the desired mutant allele. Alternatively, a direct cloning method was used, whereby the entire antibiotic resistance cassette, along with its extended upstream and downstream genomic regions was amplified as a single fragment from an existing mutant strain. This PCR product was then directly used to transform the target *B. subtilis* strain, achieving allele replacement via homologous recombination. Detailed description of strain construction was listed as follows.

SNK8123 (Δ*cotE::tet* Δ*gerE::kan*): The upstream and downstream of the mutant genes of BKK28410 and PS3328, along with the antibiotic fragment, were amplified by PCR using primer pairs gerE 3-gerE 4 and cotE 3-cotE 4, respectively. The PCR-amplified DNA was transferred into PS832, respectively.

SNK8124 (Δ*5* Δ*cotE::tet* Δ*gerE::kan*): The upstream and downstream of the mutant genes of BKK28410 and PS3328, along with the antibiotic fragment, were amplified by PCR using primer pairs gerE 3-gerE 4 and cotE 3-cotE 4, respectively. The PCR-amplified DNA was transferred into bLA201, respectively.

SNK407 (Δ*dacB::kan*): The upstream and downstream of the mutant genes of BKK23190, along with the antibiotic fragment, were amplified by PCR using primer pairs dacB up 3-dacB down 3. The PCR-amplified DNA was transferred into PS832.

SNK8126 (Δ*5* Δ*dacB::kan*): The upstream and downstream of the mutant genes of BKK23190, along with the antibiotic fragment, were amplified by PCR using primer pairs dacB up 3-dacB down 3. The PCR-amplified DNA was transferred into bLA201.

SNK8125 (Δd*acB::kan* Δc*wlD::spec*): The upstream and downstream of the mutant genes of LR48, along with the antibiotic fragment, were amplified by PCR using primer pairs cwlD up-cwlD down. The PCR-amplified DNA was transferred into SNK407.

SNK8127 (Δ*5* Δ*dacB::kan* Δ*cwlD::spec*): The upstream and downstream of the mutant genes of LR48, along with the antibiotic fragment, were amplified by PCR using primer pairs cwlD up-cwlD down. The PCR-amplified DNA was transferred into SNK8126.

SNK1171 (Δ*pssA::kan*): The upstream and downstream of the mutant genes of BKK02270, along with the antibiotic fragment, were amplified by PCR using primer pairs pssA up-pssA down. The PCR-amplified DNA was transferred into PS832.

SNK2194 (Δ*5* Δ*pssA::kan*): The upstream and downstream of the mutant genes of BKK02270, along with the antibiotic fragment, were amplified by PCR using primer pairs pssA up-pssA down. The PCR-amplified DNA was transferred into bLA201.

SNK321 (Δ*ugtP::spec*): The genomic regions upstream and downstream of the target gene were amplified by PCR using primer pairs (ugtP us up-ugtP us low, ugtP ds up-ugtP ds low). The resulting PCR products were assembled with *spec* using the Gibson Assembly method (NEB, USA). The assembly product was then used to transform PS832.

SNK325 (Δ*5* Δ*ugtP::spec*): The genomic regions upstream and downstream of the target gene were amplified by PCR using primer pairs (ugtP us up-ugtP us low, ugtP ds up-ugtP ds low). The resulting PCR

products were assembled with *spec* using the Gibson Assembly method (NEB, USA). The assembly product was then used to transform bLA201.

SNK1172 (Δ*clsA::kan*): The upstream and downstream of the mutant genes of BKK36590, along with the antibiotic fragment, were amplified by PCR using primer pairs clsA up-clsA down. The PCR-amplified DNA was transferred into PS832.

SNK2193 (Δ*5* Δ*clsA::kan*): The upstream and downstream of the mutant genes of BKK36590, along with the antibiotic fragment, were amplified by PCR using primer pairs clsA up-clsA down. The PCR-amplified DNA was transferred into bLA201.

ZQ52 (Δ*dacB::kan, amyE::PdacB-dacB-cm*): Constructed by amplifying the *dacB* gene and its promoter from gDNA of *B. subtilis* strain (PS832), using primers PdacB-up/dacB-low. The PCR-amplified DNA was cloned into pDG364 digested with BamHI and HindIII. The constructed plasimid was transformed into SNK407.

ZQ62 (Δ*5*, Δ*dacB::kan, amyE::PdacB-dacB-cm*): Constructed by amplifying the *dacB* gene and its promoter from gDNA of *B. subtilis* strain (PS832), using primers PdacB-up/dacB-low. The PCR-amplified DNA was cloned into pDG364 digested with BamHI and HindIII. The constructed plasimid was transformed into SNK8126.

ZQ63 (Δ*spmA spmB::mls*): Constructed using Gibson assembly kit (NEB, USA) to assemble PCR products, amplified with primer pairs spmAB-KO-P1/spmAB-KO-P2, spmAB-KO-P3/spmAB-KO-P4, David-universal-up/David-universal-low.

Table S2. Primers used in this study

Primer	Sequence
gerE 3	AGCGCCGGAATTTGAGTAT
gerE 4	ATACATTCCACATGCCCGA
cotE 3	TGAGAAGGCTCGCAAGTGA
cotE 4	CGTTCCTTGGCCTTGTCA
dacB up 3	CGCCGACTTTTCTTGAACA
dacB down 3	TCGGTCGTCATTCCAAGTG
cwlD up	GCTCATAATAGCAAAGCCTGG
cwlD down	GCAAAGGCACGTTTAGTTCC
pssA up	TGCGCTTTTACCAGGCAT
pssA down	CGTCTGAACCACACCGTCA
ugtP us up	CATTTCATCAGGATATCCGGC
ugtP us low	CTGAGCGAGGGAGCAGAAGTAAATTCACCTCAATGTAATCAACAA
ugtP ds up	GTTGACCAGTGCTCCCTGTGGCGTACTTGAGAGCATACG
ugtP ds low	ATCCAGCTGATGCTCGCTG
clsA up	TTGTAGCCATTTTCGTGCTG
clsA down	ATGCTGATGAGGGTTTTGTTC
PdacB-up	CCAACTGGTAATGGTAGCGACCGGCGCTCAGCGTTTACTCTCCCTTTTTCAGG
PdacB-low	GTCAAACATGAGAATTCGATAAGCTTCTAGTTATATTGACCATTTTGCTCCGC
spmAB-KO-P1	GGACATGCGCATAAACTTGTAC
spmAB-KO-P2	CTGAGCGAGGGAGCAGAATTATATTGACCATTTTGCTCCGC
spmAB-KO-P3	GTTGACCAGTGCTCCCTGAAACGGCGTTTTTTTTAGATTTGG
spmAB-KO-P4	GGACAAAATTCGGTGCGTC
David-universal-up	TTCTGCTCCCTCGCTCAG
David-universal-low	CAGGGAGCACTGGTCAAC

Reference

Amon, J. D., Artzi, L., & Rudner, D. Z. (2022). Genetic Evidence for Signal Transduction within the Bacillus subtilis GerA Germinant Receptor. *Journal of Bacteriology*, *204*(2), e00470-21. https://doi.org/10.1128/jb.00470-21

Ghosh, S., Setlow, B., Wahome, P. G., Cowan, A. E., Plomp, M., Malkin, A. J., & Setlow, P. (2008). Characterization of spores of Bacillus subtilis that lack most coat layers. *Journal of Bacteriology*, *190*(20), 6741–6748. https://doi.org/10.1128/JB.00896-08

Griffiths, K. K., & Setlow, P. (2009). Effects of modification of membrane lipid composition on *Bacillus subtilis* sporulation and spore properties. *Journal of Applied Microbiology*, *106*(6), 2064–2078. https://doi.org/10.1111/j.1365-2672.2009.04176.x

Guérout-Fleury, A.-M., Frandsen, N., & Stragier, P. (1996). Plasmids for ectopic integration in Bacillus subtilis. *Gene*, *180*(1), 57–61. https://doi.org/10.1016/S0378-1119(96)00404-0

Okada, M., Matsuzaki, H., Shibuya, I., & Matsumoto, K. (1994). Cloning, sequencing, and expression in Escherichia coli of the Bacillus subtilis gene for phosphatidylserine synthase. *Journal of Bacteriology*. https://doi.org/10.1128/jb.176.24.7456-7461.1994

Perkins, D. L., Lovell, C. R., Bronk, B. V., Setlow, B., Setlow, P., & Myrick, M. L. (2005). Fourier transform infrared reflectance microspectroscopy study of Bacillus subtilis engineered without dipicolinic acid: The contribution of calcium dipicolinate to the mid-infrared absorbance of Bacillus subtilis endospores. *Applied Spectroscopy*, *59*(7), 893–896. https://doi.org/10.1366/0003702054411742

Popham, D. L., Illades-Aguiar, B., & Setlow, P. (1995). The Bacillus subtilis dacB gene, encoding penicillin-binding protein 5*, is part of a three-gene operon required for proper spore cortex synthesis and spore core dehydration. *Journal of Bacteriology*, *177*(16), 4721–4729. https://doi.org/10.1128/jb.177.16.4721-4729.1995

Popham, D. L., Meador-Parton, J., Costello, C. E., & Setlow, P. (1999). Spore Peptidoglycan Structure in a cwlD dacB Double Mutant of Bacillus subtilis. *Journal of Bacteriology*, *181*(19), 6205–6209. https://doi.org/10.1128/jb.181.19.6205-6209.1999

Schottroff, F., Pyatkovskyy, T., Reineke, K., Setlow, P., Sastry, S. K., & Jaeger, H. (2019). Mechanisms of enhanced bacterial endospore inactivation during sterilization by ohmic heating. *Bioelectrochemistry*, *130*, 107338. https://doi.org/10.1016/j.bioelechem.2019.107338

Setlow, P. (2007). I will survive: DNA protection in bacterial spores. *Trends in Microbiology*, *15*(4), 172–180. https://doi.org/10.1016/j.tim.2007.02.004

Setlow, P., & Christie, G. (2021). What's new and notable in bacterial spore killing! *World Journal of Microbiology & Biotechnology*, *37*(8), 144. https://doi.org/10.1007/s11274-021-03108-0
